# Atmospheric Deposition of Local Mineral Dust Delivers Phosphorus to the Greenland Ice Sheet

**DOI:** 10.1021/acs.est.5c13873

**Published:** 2026-01-13

**Authors:** Jenine McCutcheon, James B. McQuaid, Nuno Canha, Sarah L. Barr, Stefanie Lutz, Vladimir Roddatis, Sathish Mayanna, Andrew J. Tedstone, Martyn Tranter, Liane G. Benning

**Affiliations:** † School of Earth & Environment, 150405 University of Leeds, Woodhouse Lane, Leeds LS2 9JT, U.K.; ‡ Department of Earth and Environmental Sciences, University of Waterloo, Waterloo, ON N2L 3G1, Canada; § HyLab - Green Hydrogen Collaborative Laboratory, Estrada Nacional 120-1 Central Termoeléctrica, Sines 7520-089, Portugal; ∥ 28337 GFZ, Helmholtz Centre for Geosciences, Telegrafenberg, Potsdam 14473, Germany; ⊥ Department of Plant and Microbial Biology, University of Zürich, Zurich 8008, Switzerland; # Carl Zeiss Microscopy GmbH, Carl Zeiss Strasse 22, Oberkochen 73447, Germany; ∇ Bristol Glaciology Centre, 1006 University of Bristol, Bristol BS8 1SS, U.K.; ○ Department of Geosciences, Université de Fribourg, Fribourg 1700, Switzerland; ◆ Institute of Earth Surface Dynamics, University of Lausanne, Lausanne 1015, Switzerland; ¶ Department of Environmental Science, Aarhus University, Frederiksborgvej 399, Roskilde 4000, Denmark; 11 Department of Earth Sciences, Freie Universität Berlin, Berlin 12249, Germany; 12 National Centre for Atmospheric Science, University of Leeds, Leeds LS2 9PH, U.K.

**Keywords:** Greenland ice sheet, mineral dust, aerosols, snow, grain size, nutrients, glacier ice algae, phosphorus

## Abstract

Aerosol composition, size, and deposition rate determine the impact these particles have on cryosphere environments. Mineralogical, biological, and geochemical characteristics of aerosols collected over two years from the southwest Greenland Ice Sheet indicate that aerosols delivered via dry deposition and in snow primarily consisted of silicate minerals, with mean particle diameters of 1.01 ± 1.58 μm (2016) and 0.76 ± 0.87 μm (2017) for dry deposition and 2.4 ± 3.2 μm for dust delivered in snow (2017). The rare earth element signature of the delivered dust was typical of nearby Greenlandic lithologies, and combining this with other geochemical results and airmass history modeling indicated that the airborne mineral dust collected *on-ice* was likely from local emission sources, namely nearby proglacial plains. Dust and snow deposition rates were used to estimate phosphorus delivery to the ice surface at a rate of 1.2 mg·m^–2^·year^–1^, which could fuel estimated pigmented glacier ice algal cell abundances of 8.6 × 10^3^ cells·mL^–1^, a value consistent with glacier ice algal bloom cell densities documented in the region. The eukaryotic communities in air and snow samples were dominated by algae and fungi, respectively, with both sample types also hosting various bacteria. These results suggest that the airborne transfer of glacier ice and snow algae may be a method by which fresh cryosphere surfaces become inoculated with these pigmented organisms. Collectively, these findings highlight the biogeochemical links between aerosols and the ice sheet surface, which have impacts on glacier ice algal growth and the corresponding surface ice albedo and melting.

## Graphical abstract

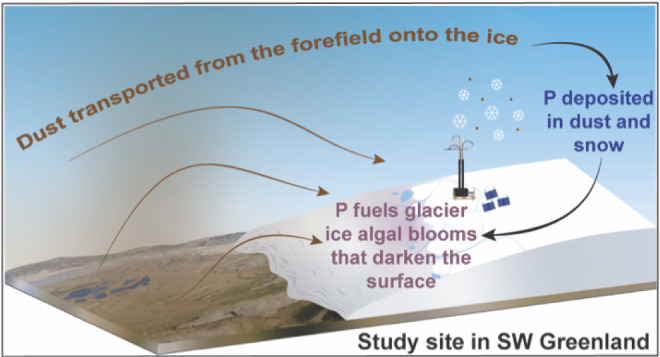

## Introduction

The rate of ice mass loss from the Greenland Ice Sheet (GrIS) has accelerated in recent years, with approximately half of the measured loss due to meltwater discharge^1^. Surface melting of the GrIS is controlled by the flux of incoming shortwave radiation,^2,3^ modulated by ice albedo.^4^ Albedo is a product of surface ice structure and the type, abundance, and distribution of light-absorbing particulates (LAPs).^5,6^ Abiotic LAP, such as black carbon (BC; soot) and mineral dust,^7,8^ and biological LAP, such as pigmented snow^9^ and glacier ice algae,^10,11^ can individually or collectively reduce snow and ice albedo. The impact of mineral dust on snow and ice albedo is variable and is influenced by factors, including dust concentration and optical properties, snow and ice physical properties, and the manner by which dust is mixed into the snow or ice.^12^ The flux of dust deposited in Greenland has been estimated to be 0.2 Tg·year^–1^.^12^ Dust emitted from high-latitude sources, including proglacial regions of Greenland, is important because it makes up an estimated 57% of the dust deposited on Arctic snow- and ice-covered regions,^13^ yet local polar dust sources are not well constrained by on-the-ground measurements. Emission of fine-grained glacial dust from proglacial plains and subsequent local and regional deposition are particularly important in Greenland, with notable dust sources^14,15^ found near some of the fastest-melting parts of the GrIS, including the location studied here. We have recently^16^ demonstrated that mineral dust in surface ice habitats in the “Dark Zone” on the southwestern margin of the GrIS provides phosphorus to the surface ice microbial community, fueling glacier ice algal blooms and associated albedo reduction.^16^ Glacier ice and snow algae grow on melting cryosphere surfaces and impact ice and snow albedo and melting.^17–19^ Glacier ice algae are responsible for as much as 13% of the runoff generated from the bare-ice regions in the southwest drainage basin of the GrIS during recent melt seasons,^5,20^ while red-pigmented snow algae can reduce local albedo by 13%.^9^ If mineral dust provides nutrients to these organisms, then it is important to understand dust composition and deposition in the context of biogeochemical nutrient cycling in supraglacial habitats.

Our aim was to characterize the geochemistry, mineralogy, grain size, and microbiology of particulates in snow, ice, and aerosol samples collected at a study site located ∼35 km from the western GrIS margin in the Dark Zone, during the early (June 2017) and mid to late (July–August 2016) stages of the annual melt season. We provide the first mineral dust delivery rates estimated for dry and wet (via snow) deposition for an *on-ice* location in the southwestern GrIS. By connecting mineral dust geochemistry and size measurements to measured local meteorology and atmospheric transport using airmass hindcast modeling, we gained a better understanding of local dust transport and deposition. These data also allowed us to estimate the total delivered phosphorus in dust and snow and to calculate the corresponding potential glacier ice algae abundance and primary production. Together, these findings provide insight into nutrient delivery and cycling on the GrIS and have implications for ice albedo and mass loss.

## Materials and Methods

### Study Location and Aerosol Sampling

Aerosol monitoring and sample collection were conducted at a site (67.07° N, 49.38° W, 1073 m AMSL) in the ablation zone in southwest Greenland (Figure 1). Sampling and monitoring took place from July 12 to August 18, 2016, and June 1 to 28, 2017, during the spring and summer melt period. A 2 m tall sampling mast was erected 50 m upwind of all camp structures to support the aerosol sampling instruments. Aerosol particulates were sampled onto 47 mm diameter polycarbonate filters (0.22 μm pore size). The filters were housed in unheated, inverted PFA filter holders (Savillex), modified for open path sample collection, and mounted on the sampling mast. Air was sampled onto these filters at 3.8–10 L·min^–1^ (variation due to power fluctuations) for exposure times ranging between 11 and 48.5 h per filter. Aerosols were collected in 2016 (*n* = 7 filters) and 2017 (*n* = 4 filters), and all filters were stored in PetriSlides until analysis (see (Supporting Information SI) Table S1,S2). A Bertin Instruments Coriolis μ air sampler was used to collect larger volume “snapshots” of airborne particulates above the ice surface on June 6, 10, 11, 13, 14, 15, 16, and 28 in 2017. Volumes (6,000–54,000 L) of air were sampled 60–70 cm above the snow or ice surface over short periods (20 min–3 h) into 15 mL of sterile deionized water at 300 L·min^–1^. The water was filtered onto 0.22 μm polycarbonate filters, and the filters were stored in PetriSlides (Table S2).

**Figure 1:**
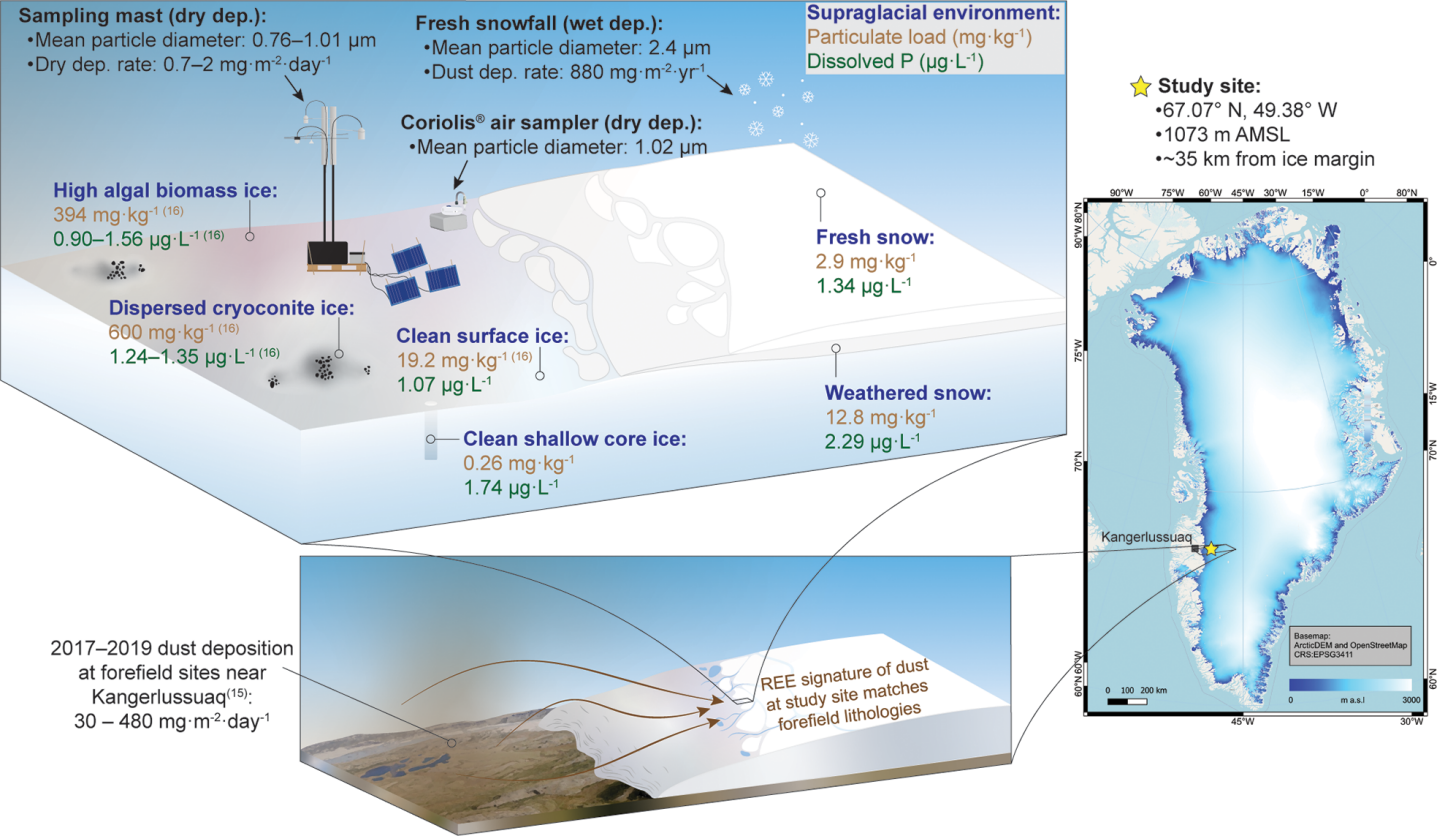
Location of the study site in southwest Greenland (star, right panel), where particulate loads (brown text; mg·kg^–1^) and dissolved phosphorus concentrations (green text; μg·L^–1^) were measured in various supraglacial environments (named in blue text). Aerosol particle diameters are given for each of the airborne sample types (black text). Rates of dry and wet deposition (in snow) of dust were calculated (black text) and compared to forefield dust deposition rates measured near Kangerlussuaq in 2017–2019 ^15^ (lower left panel). The dust collected at the site contained a rare earth element signature similar to forefield lithologies; dust from the forefield is shown being transported onto the ice (lower left panel). Values presented as ranges represent multiple seasons, and values presented as single values are from 2017. Some surface ice particulate and phosphorus values are from McCutcheon et al.^16^ The Greenland base map was generated using Digital Elevation Models (DEMs)^65^ as part of the Greenland Ice Mapping Project (GIMP).^66^

An Alphasense OPC-N2 optical particle counter (OPC) mounted on the mast was used to measure aerosol particle size distribution in 2016 (16/07/2016–01/08/2016) and 2017 (11/06/2017–28/06/2017). The measurements were made at 1 min time intervals and a total flow rate of 0.22 L·min^–1^. Additional instrument and data processing details are described in the SI file.

### Scanning Electron Microscopy (SEM)

Subsections (∼8 × ∼8 mm) of the polycarbonate filters were cut out using a razorblade and mounted on aluminum stubs using adhesive carbon tabs. The samples were coated with 5 nm of iridium using an Agar High-Resolution sputter coater. Automated particle imaging was conducted using a Zeiss Ultra Plus Field Emission SEM operated at 15 kV and imaged using a BSD (backscattered detector) with automated Zeiss Smart-Stitch software. Counting and size distribution measurements of the BSD overview images were calculated and processed using ImageJ.^21^ For each particle, size is expressed as a diameter (μm) calculated for a sphere with an area equal to the two-dimensional particle surface area visible in the XY plane in the microscope. Particle size analysis was conducted on filters from 2016 (*n* = 7 filters, *n* = 4,466 particles measured) and 2017 (*n* = 4 filters, *n* = 14,070 particles measured). The presented particle size data are cutoff at an upper size limit of 17 μm to match the OPC data, with particles >17 μm in diameter accounting for <0.01% of the measured particles. Note that the difference in the average particle size between the 2016 and 2017 sample years may have been impacted by the different respective sample sizes, but the sample sizes are suitable for comparison purposes.^22^ Elemental mapping using energy dispersive spectroscopy (EDS) to determine bulk dust elemental composition and additional electron microscopy were conducted as described in the SI file.

### Snow and Ice Core Sampling

The ice surface was free of snow during the 2016 field campaign, thus preventing snow sampling that year. At the beginning of the 2017 campaign, the ice was covered with winter snow accumulation, and we sampled this “weathered” snow as well as “fresh” snow from two snowfall events (June 17 and June 20, 2017) during the field season, each of which generated 2–5 cm of accumulation. Fresh snow was removed from the underlying snow crust or bare ice using sterile plastic scoops and transferred to sterile plastic bags, in which the samples were melted at ambient temperature (0–10 °C). Particulates in melted snow samples of known volume were filtered onto preweighed 5.0 and 0.22 μm pore-size polycarbonate filters, which were dried and reweighed to quantify the particulate mass. Filters were weighed using a Sartorius ME5 Microbalance mass balance (readability: 1 μg, repeatability: ± ≤ 1 μg) in conjunction with an antistatic gun.^23^ Samples (*n* = 8) of weathered snow remaining from winter accumulation were collected in the same manner. To sample shallow ice, a preconditioned 14 cm ice corer (Kovacs) was used to collect ∼1 m cores of homogeneous ice that was free of any macroscopically visible particulate matter. The cores were placed onto aluminum foil (ashed at 550 °C for 8 h) and cut into ∼30 cm long segments using a clean stainless steel saw. The cores were melted, and particulate matter was collected onto polycarbonate filters in the same manner as the snow samples.

### Snow Dust Analyses

The particle size distribution was measured for the particulates collected out of fresh (*n* = 1) and weathered (*n* = 2) snow samples. The sample number was limited due to the amount of material required for analysis. This analysis was completed using a DC24000 CPS disc centrifuge^24^ at Oxford Materials Characterization Services, Oxford, UK. Mineral dust collected on polycarbonate filters (pore size: 0.22 μm) from melted fresh and weathered snow was characterized using Rietveld refinement of X-ray diffraction data (XRD). Samples were milled using an agate mortar and pestle prior to loading in 5 mm low-background silicon mounts. Shallow sample mounts were required due to the small quantity of material per sample. The samples were, therefore, not infinitely thick with respect to X-rays, rendering the results semiquantitative. The analysis was conducted using a Bruker D8 Advance Eco X-ray diffractometer (Bruker, Billerica, USA) with a Cu source, operated at 40 kV and 40 mA (see SI for details). Rare earth element (REE) sample preparation and analysis were conducted on snow samples (*n* = 3) in the same manner as described in McCutcheon et al.,^16^ using HR-ICP-MS (ThermoFisher Element 2). Precision for all elements was better than 2% RSD (see the SI for details).

### Snow Meltwater Chemistry

Samples of melted snow and ice were filtered using 0.22 μm single use syringe filters and acidified using Aristar HNO_3_ prior to the measurement of cation concentrations using inductively coupled plasma mass spectroscopy (ICP-MS; Thermo Fisher iCAPQc, see SI for analytical details). For melted samples, conductivity and total dissolved solids (TDS) were measured using an Orion Star A222 m, and pH was measured using an Orion Star A321 m (Table S9).

### Microbial Community Composition

The microbial community composition in air (2016 and 2017) and snow (2017) samples was determined using 16S and 18S amplicon sequencing as described in the SI file, similar to the method used in McCutcheon et al.^16^ Air samples were collected into Milli-Q water using the Coriolis μ air sampler prior to filtration using sterile Nalgene single-use filtration units (pore size: 0.22 μm). The filters were transferred to 5 mL cryo-tubes and immediately frozen in a cryo-shipper at liquid nitrogen temperatures, and upon return to the home laboratory, the samples were stored at −80 °C until processed. Each sample collected for community composition analysis was paired with a sample for particulate analysis (Table S1,S2).

### Dry Deposition Velocity and Flux Calculations

Particle deposition was estimated as a function of particle size and wind speed by using the method developed by Hoppel et al.^25^ for particle deposition for a uniform surface in equilibrium. See the SI file for detailed methods.

### Meteorology

A WS-GP1 weather station (Delta T Devices Ltd.) was used at the site during the 2017 campaign to monitor air temperature, relative humidity, incident solar radiation (proxy for cloud cover), wind speed, and wind direction at 1 min intervals. The 2017 air pressure data are from the nearby S6 automatic weather station (67°04′ N, 49°23′ W, ∼1000 m AMSL), operated by the Utrecht University Institute for Marine and Atmospheric Research (IMAU), which generated data at 30 min intervals.^26^ All of the 2016 meteorological data are from the S6 weather station due to meteorological data at our study site being unavailable. A comparison between our 2017 onsite meteorological measurements and the S6 weather station confirms that the data from S6 are representative of our study site. Outputs from the regional climate model MAR v3.11.2 at 6 km resolution, forced by ERA5 reanalysis, were used to estimate snowfall rates at the study location.^27^

### Airmass History Modeling

The history of sampled airmasses was investigated using the hindcast Lagrangian FLEXible PARTicle Dispersion Model (FLEXPART) in order to infer aerosol transport.^28^ FLEXPART was run in backward mode, driven by ERA5 reanalysis data,^29,30^ with simulations initiated every 6 h during the 2016 and 2017 campaign periods. In each simulation, 10000 particles were released from the surface at the sampling site location (67.07° N, 49.38° W, 1073 m AMSL) and followed backward for 10 days. The transport of simulated particles considers the average flow as well as turbulent and diffusive transport, convection, and loss processes such as wet and dry deposition. The output is a grid of potential emission sensitivities (PES) at 3-hourly timesteps, as well as a back trajectory calculated from the average particle position at each timestep. PES represents the residence time of particles in a grid cell and is proportional to the contribution a source in the grid cell would make to the mass concentration at the particle release location (the sampling site). The output of each 10-day simulation was integrated over all timesteps to create a total PES plot every 6 h. Since emissions from dust sources at the surface are of interest, these PES plots show emission sensitivities from 0 to 2500 m above ground level (AGL). In addition, simulations from the two campaign periods were aggregated to create PES plots for the 2016 and 2017 measurement campaigns.

## Results and Discussion

### Characterization of Dry-Deposited Mineral Dust

Electron microscopy of the airborne particulates collected on filters indicated that mineral dust was the dominant aerosol present (Figure 2a). The dust grains were submicrometer to tens of micrometers in diameter, with mean particle diameters of 1.01 ± 1.58 and 0.76 ± 0.87 μm for 2016 and 2017 samples, respectively (Figures 1, 2b and S1; Table S2). Over 99% of the >18,500 particles measured were <5 μm in diameter. SEM measurements of aerosols collected from the large volume air samples yielded a mean particle diameter of 1.02 ± 1.57 μm (*n* = 51,484), with 98% of the particles <5 μm in diameter (Figures 1, 2c; Table S2). The dust particles are mainly composed of Si and O and also contain Al, Na, Mg, Fe, and Ca, suggesting that they correspond to silicate minerals (Figure 2d, S2; Table S3). Within the characterized dust were multiple visibly weathered grains containing collocated Ca and P (Figure 2e–g, S3), which were assigned to the mineral apatite/hydroxylapatite (Ca_5_[PO_4_]_3_[Cl/F/OH]). These grains demonstrate that phosphorus is delivered to the ice sheet surface via airborne deposition, which has important implications because phosphorus is an essential nutrient for the glacier ice algal blooms that are responsible for albedo reduction and accelerated surface ice mass loss at the site.^5,16,20,31–33^ A small number of other particulate types beyond mineral dust were observed on the filters, including cells inferred to be glacier ice algae, pollen, and soot particles (Figure 2h–k). These constituents were far less abundant than mineral dust, but indicate the presence of trace airborne organic and inorganic carbonaceous matter over the GrIS. Salt crystals were not observed on the filters, suggesting that dissolved solutes were not major constituents in the collected aerosols.

**Figure 2:**
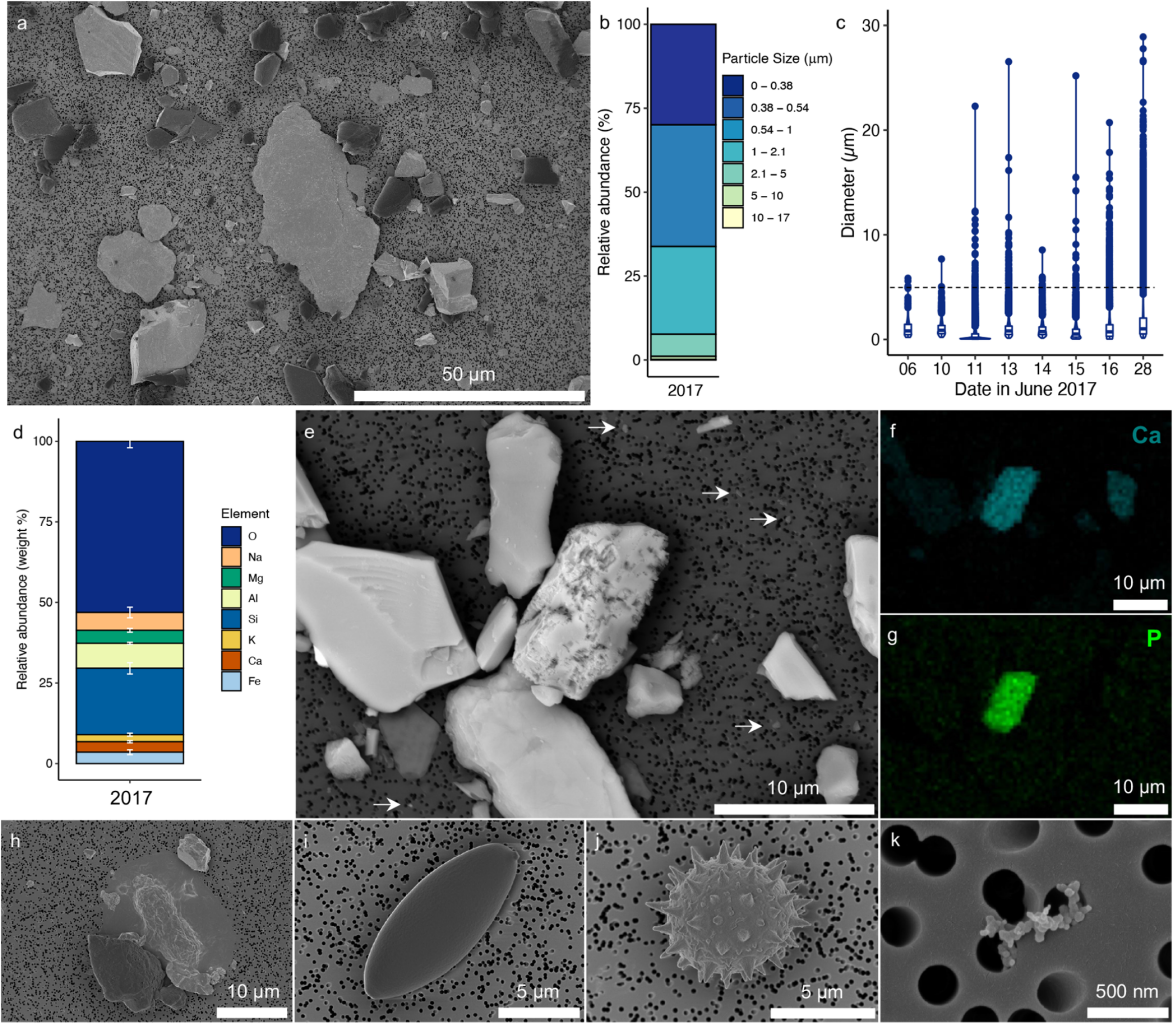
2017 a) Backscattered electron (BSE) micrograph showing the mineral dust on the aerosol filters; b) size distribution of the mineral dust collected on filters in 2017 (*n* = 4; particles: *n* = 14,070; see Figure S1 for 2016 data); c) violin and box plot showing the particle size distribution for mineral dust collected using a Coriolis μ air sampler in June 2017 (see Table S2 for sample details), with the black dashed line indicating 5 μm; d) SEM-EDS measured elemental composition of mineral dust grains in the June 28, 2017 Coriolis sample (GrIS-17-CR-16) expressed as weight % (total area analyzed = 7.1 mm^2^); e) BSE micrograph showing the size range of the mineral dust grains in the June 28 Coriolis sample (<1 μm diameter grains indicated by arrows); and a highly textured grain that contained collocated f) Ca and g) P (see Figure S3 for more examples). The aerosol samples contain a few nonmineral particles, including h) and i) cells presumed to be algal cells, j) pollen, and k) soot particles.

### Characteristics of Mineral Dust in Surface Snow and Shallow Ice

Electron microscopy of particulates collected from the weathered snow revealed that these were also predominantly comprised of mineral dust grains <1 μm in diameter (Figure 3a,b). The size distributions of particulates measured in the fresh and weathered snow samples, presumed to be mostly mineral dust based on the microscopy results, were very similar, with an overall mean of 2.4 ± 3.2 μm (*n* = 10,289 particles measured) (Figures 1, 3d). As much as 15% of the particulates were >5 μm in diameter, indicating that snow delivers proportionally larger dust grains compared to dry deposition.

**Figure 3:**
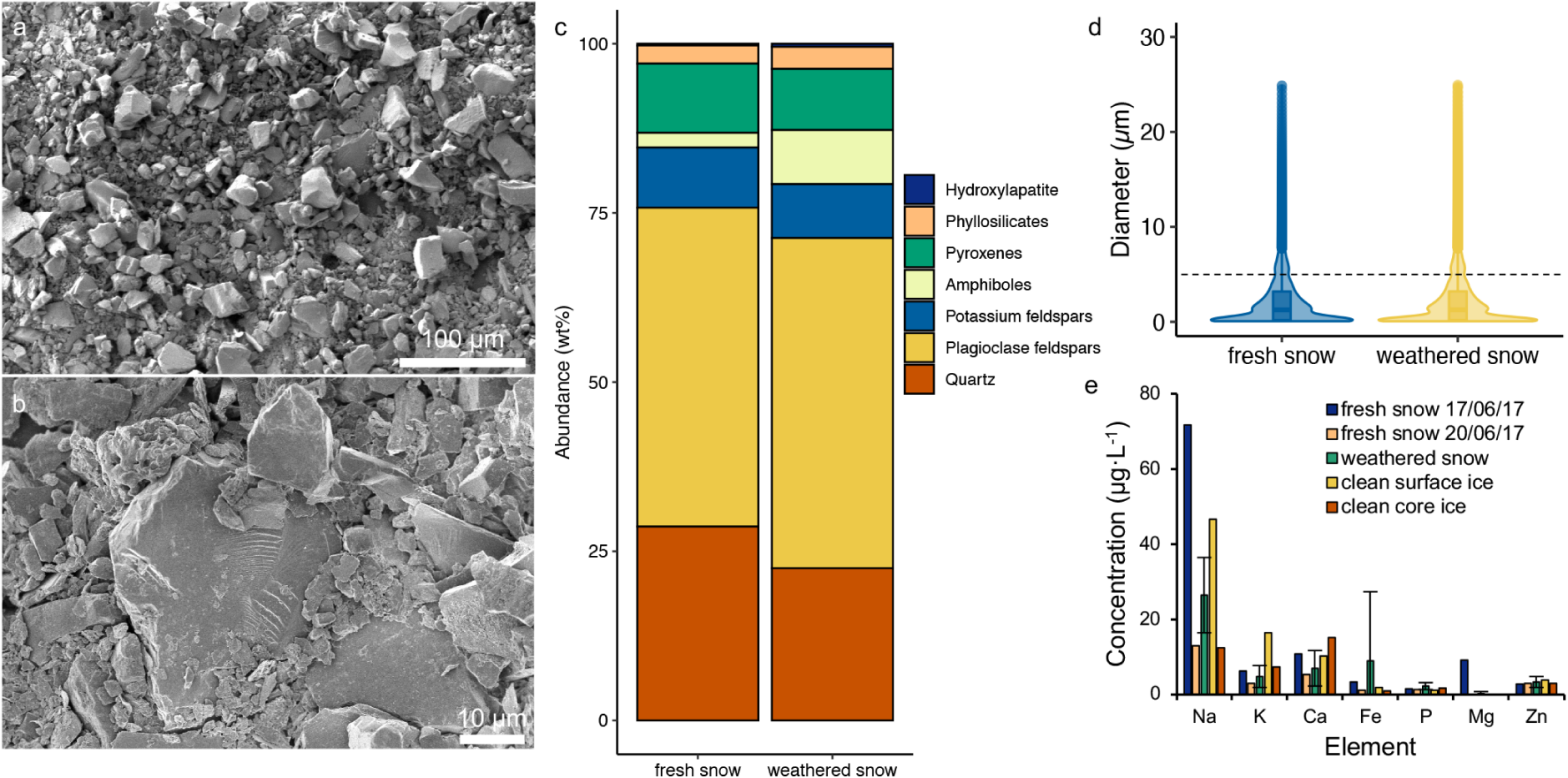
(a, b) SE-SEM micrographs showing morphology of the abundant mineral dust grains collected from snow; (c) mineralogy of the mineral dust in the fresh and weathered snow; (d) violin and box plot showing the particle size distribution for the mineral dust collected in the fresh (*n* = 1) and weathered (*n* = 2) snow samples, with the black dashed line indicating 5 μm; and (e) concentrations of major dissolved ions measured in fresh snow (*n* = 2), weathered snow (*n* = 5), clean surface ice (*n* = 1), and clean core ice (*n* = 1) samples collected at the campaign site in 2017. See Tables S7 and S8 for detailed meltwater chemistry.

The mineralogical composition of the dust collected from fresh and weathered snow consisted predominantly of framework silicates (e.g., feldspars and quartz), phyllosilicates, and inosilicates (Figure 3c, Table S5). Despite dust quantities recovered from snow samples suitable for mineralogical characterization being limited, the mineralogical composition was comparable to dust accumulated in cryoconite holes and on the bare ice sheet surface at this location and in the same study years^16^ and is compatible with the bulk elemental composition of the dust collected on the aerosol filters (Figure 2d). For a comparison more broadly to other GrIS sites, the mineralogy of the dust in the snow is also similar to that in ice cores from the East Greenland Ice-core Project (EGRIP), which was similarly rich in silicate minerals.^34^ Minor quantities of hydroxylapatite (0.3 wt % in fresh snow) were also present, corroborating the aerosol sample SEM-EDS findings (Figure 2e–g). Aerosol deposition of phosphorus has been documented in other cryosphere environments; measurements at a site in the Himalayas indicated that mineral dust accounted for 77% of the total phosphorus in aerosols.^35^ While the phosphorus delivery in aerosols will vary with the mineral dust source, a calculation of estimated phosphorus delivery to the ice surface via atmospheric deposition based on our collected data is presented below.

The REE signature of the mineral dust in the fresh and weathered snow shows a positive Eu/Eu* anomaly (Figure S4, Table S6) and is similar to REE signatures of mineral dust accumulated on the bare ice surface at the site,^16^ signatures from nearby cryoconite minerals,^36^ and REE signatures of local Greenlandic rocks and sediments.^16,37,38^ This result suggests that mineral dust delivered to the study site in snow was primarily derived from local lithologies. A local dust provenance is consistent with data from deep ice cores collected at other locations near the GrIS margin (e.g., Hans Tausen and Renland).^39^ In contrast, deep ice cores from more central GrIS sites (e.g., Dye 3, Site A, GRIP, and NorthGRIP) typically contain dust with a signature more closely matching that of East Asian desert mineral dust.^39^

With respect to particulate mass loading, fresh snow contained 2.9 mg·kg^–1^ (mean, *n* = 2) of particulate matter (Table S4), approximately four times less than weathered snow (12.8 ± 4.5 mg·kg^–1^, *n* = 8; Figure 1, Table S4). These values may suggest variability in dust load and/or that mineral dust in snow becomes concentrated with partial melting after snow deposition. Sample numbers for fresh snow were limited by the number of snowfall events during the field campaign, and future studies would benefit from more extensive sampling of fresh snow to better constrain these particulate loading values and the corresponding mineralogy. We previously reported a dust concentration of 19.2 ± 12.3 mg·kg^–1^ (*n* = 4) for clean ice surface samples from the same site and sample years,^16^ suggesting accumulation of dust on the ice surface following snowmelt. Our snow dust concentrations are higher than those measured in surface snow inland from Qaanaaq in NW Greenland (0.1–1.3 mg·kg^–1^).^40^ However, the forefield proximal to our *on-ice* study site near Kangerlussuaq is one of the largest ice-free regions in Greenland and a well-known dust source,^14,15^ while Qaanaaq lacks comparable dust sources. Airborne dust data from various forefield locations near Kangerlussuaq indicated that spring 2017 was a particularly “dusty” season,^15^ with measured dust deposition rates up to an order of magnitude higher (480 mg·m^–2^·day^–1^) than those measured during all other seasons (2017–2019) in that study (30–70 mg·m^–2^·day^–1^).^15^

We estimated dust melt-out from clean ice during seasonal ablation using shallow ice cores sampled from below the seasonally refrozen weathering crust. Ice cores contained a mean particle mass load of 0.26 mg·kg^–1^ (*n* = 2) (Figure 1), a dust load value that is similar to dust concentrations (∼0.1–1 mg·kg^–1^) reported for the NGRIP and RECAP deep ice cores.^41^ Annual surface ablation at site S6 was measured as 1.99 and 1.12 mWE in 2016 and 2017, respectively.^42^ Combining these ablation rates with the measured ice core particulate concentration yielded particulate melt-out rates of 517 and 291 mg·m^–2^·year^–1^ for 2016 and 2017, respectively.

Mineral dust and other particulate matter that accumulate on the ice surface may be retained, redistributed on the surface, or lost from the surface ice habitat in supraglacial meltwater. Mineral dust retention is common in surface ice hosting high concentrations of glacier ice algae, with these patches of ice often containing an order of magnitude more mineral dust than adjacent patches of “clean” ice. We previously documented that mineral dust accounted for 94% of 394 ± 336 mg·kg^–1^ (*n* = 3) particulates measured in high algal biomass ice at this site,^16^ and this retention of dust becomes important if it is providing nutrients to the surface microbial community.

### Snow and Ice Core Meltwater Chemistry

Quantifying solutes in the melted snow and ice samples indicated low concentrations of dissolved major ions (Figures 1, 3e, and Table S7), indicating no net effect from preferential retention or elution of dissolved cations. All samples contained low concentrations of dissolved phosphorus, namely, fresh snow (1.34 μg·L^–1^, *n* = 2), weathered snow (2.29 μg·L^–1^, *n* = 5), clean surface ice (1.07 μg·L^–1^, *n* = 1), and clean shallow core ice (1.74 μg·L^–1^, *n* = 1) (Figure 1, Table S8). These concentrations are similar to values we reported for clean surface ice in 2016 (0.37 μg·L^–1^, *n* = 3), high algal biomass ice in 2016 (0.90 μg·L^–1^, *n* = 5) and 2017 (1.56 μg·L^–1^, *n* = 3), and dispersed cryoconite ice in 2016 (1.24 μg·L^–1^, *n* = 3) and 2017 (1.35 μg·L^–1^, *n* = 1).^16^ The phosphorus concentrations measured in snow and ice are also similar to those of previous analyses of clean surface snow from Greenland^33,43^ and in deeper inland ice cores.^44^ Results of meltwater pH, conductivity, and TDS measurements can be found in Table S9.

### Microbial Community Composition in Air and Snow

The microbial community in air (*n* = 10) and snow (*n* = 4) samples was characterized to better connect *on-ice* biogeochemistry with local atmospheric processes. After quality filtering of the microbial community data, the number of reads per sample ranged between 7,160 and 81,694 and 5,471 and 134,618 for 16S and 18S amplicon sequencing, respectively. The bacterial community composition was reasonably consistent across both years and between the two habitats (air and snow), with Proteobacteria being the dominant phylum, followed by Bacteroidota (Figure 4a). In contrast, the eukaryotic community composition showed distinct habitat-related differences: air samples were enriched with algal phyla (Chlorophyta, Ochrophyta, and Phragmoplastophyta), whereas snow samples contained higher abundances of fungal phyla (Ascomycota and Basidiomycota) (Figure 4b). The fresh and weathered snow samples were reasonably similar to each other, except for one weathered snow sample containing notably high Tardigrada. The algal community was primarily composed of the glacial ice alga, *Ancylonema nordenskioeldii*, in the 2016 air and 2017 snow samples (Figure 4c). In contrast, the 2017 air samples were dominated by the snow alga *Chlamydomonas nivalis* and the cosmopolitan green alga *Desmococcus olivaceus*. The bacterial and fungal phyla identified in the air and snow samples are consistent with previous results from snow and ice samples from the area.^16,45^ The prominence of *A. nordenskioeldii* in the algal community is consistent with ice samples that we analyzed from the site in the same seasons.^16^ The differences in air sample algal community composition between 2016 and 2017 may be due to the timing of the sampling. Specifically, the sampling in 2016 occurred later in the melt season when extensive glacier ice algal blooms had developed on the ice surface. These blooms are typically dominated by *A. nordenskioeldii* and *Ancylonema alaskana*.^10^ In contrast, the 2017 sampling occurred at the onset of the melt season when much of the ice was still covered in snow containing snow algae and preceded extensive glacier ice algal bloom development such as that observed in 2016. Seasonal differences in aerobiology have been documented in other cryosphere environments, with air and snow microbial community composition changing by season.^46^ In the context of this study, the airborne microbial community may similarly evolve with the progression of the melt season and the transition of the surface covering from snow to bare ice. Airborne transport of microbial communities has been documented in polar desert and ice-covered environments and is of interest as a pathway for organism dispersion and inoculation of new surfaces.^47–49^ Identification of glacier ice and snow algae in the air samples at our study site indicates that at least some airborne dispersion of microbes is occurring above the ice sheet surface, which may have implications for the inoculation of fresh snow and ice surfaces.

**Figure 4:**
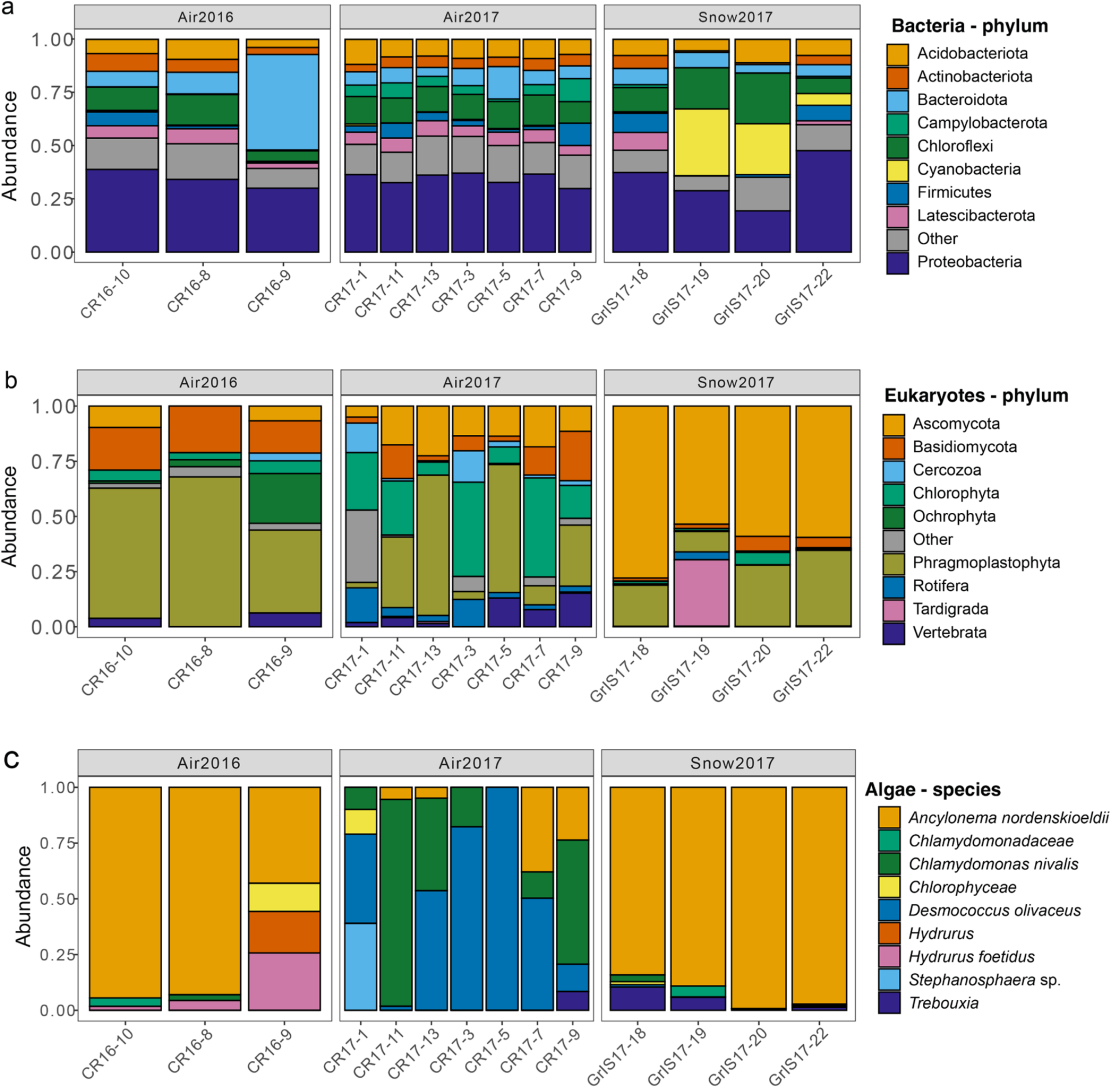
Relative abundance plots for a) bacterial phyla, b) eukaryotic phyla, and c) algal species. In (a) and (b), the nine most abundant taxa are shown, with the remainder compiled under “Other”.

### Estimation of Dry and Snow Deposition Rates for Mineral Dust

Results of time-resolved aerosol particle size measurements using the OPC matched the microscopy of the aerosols collected on filters and confirmed that particles <1 μm in diameter were the most abundant. In 2017, 84.1% and 99.6% of the particles measured by the OPC (*n* = 14,385) were <1 μm and <5 μm in diameter, respectively, with the same size fractions accounting for 93.6% and 99.9% of the particles from the 2016 sampling period (*n* = 10,791 measurements) (Figure 5a, S5). Based on the microscopy observations, we presume that the particles measured by the OPC were predominantly mineral dust. The OPC measurements were used to calculate the particle load per volume of air and estimate a dry deposition particle flux as a function of the particle diameter (Figure 5b, S5). Comparison of the 2017 OPC particle counts and particle flux indicates that large particles (>10 μm diameter), although few in abundance (<0.1–0.4%), contributed the majority of the total mass. Dry deposition rates for 2017 and 2016 were calculated as 2.0 and 0.77 mg·m^–2^·day^–1^, respectively. Comparing these *on-ice* dry deposition estimates to those measured off-ice by van Soest et al.^15^ in spring of 2017 (480 mg·m^–2^·day^–1^) indicates that only a small fraction of the airborne dust in the Kangerlussuaq forefield is transported inland to our study location. Extrapolating our daily estimates to annual dry deposition values yields rates of 735 and 282 mg·m^–2^·year^–1^, respectively. However, such values must be interpreted with caution since they are based on data from temporally constrained sampling periods during the spring and summer months, and dry deposition rates vary between seasons and with meteorological events. Nevertheless, they provide some indication of the airborne mineral dust over this region of the GrIS. Multiseason, *on-ice* measurements of aerosols would aid in constraining representative dry deposition rates, as well as capturing deposition variability caused by short, but important, dust delivery events.

**Figure 5:**
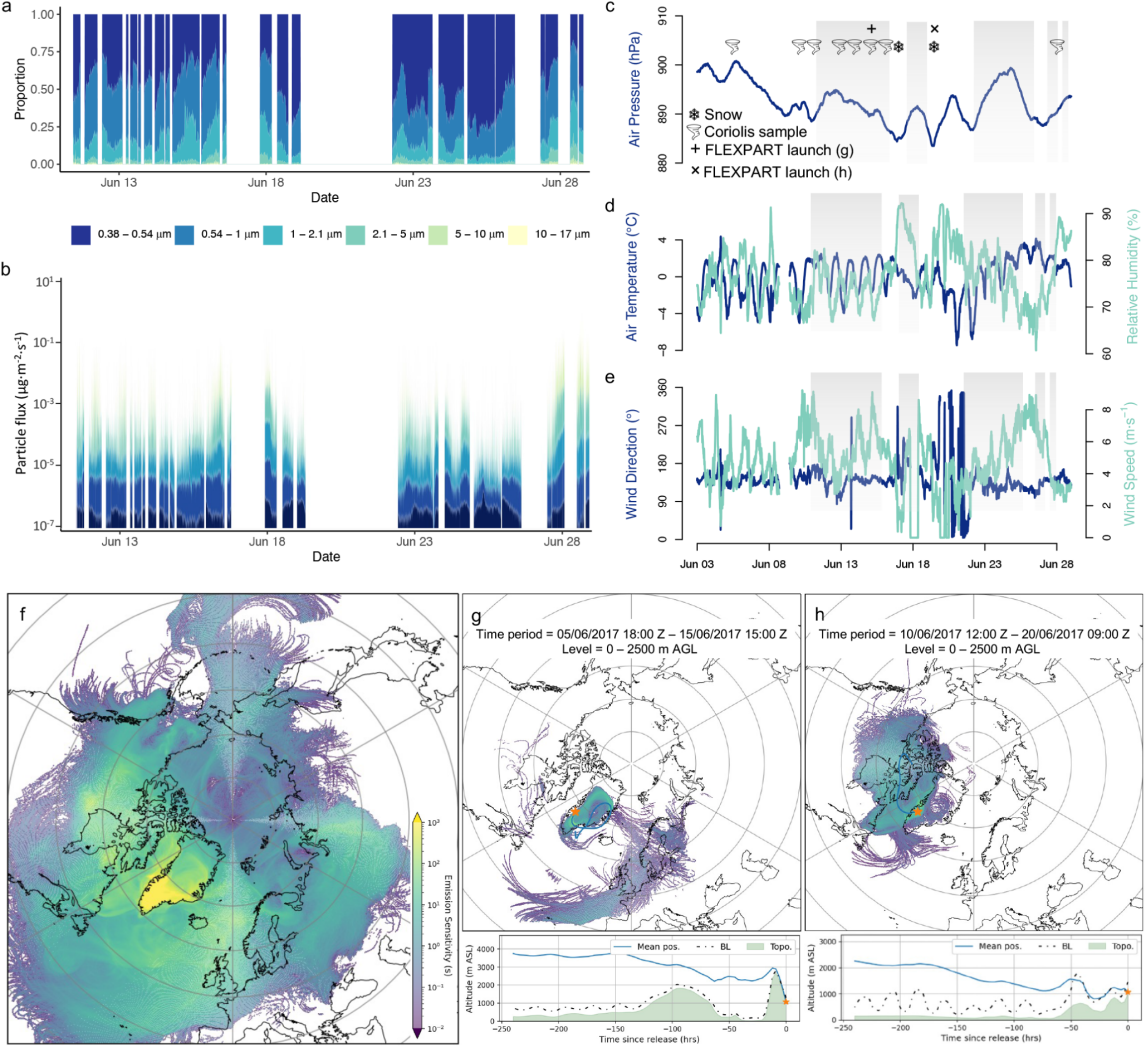
2017 campaign time-resolved: a) proportion of particles by size bin measured using the OPC; b) particle flux by size bin; and meteorological measurements of c) air pressure, d) air temperature and relative humidity, and e) wind speed and direction. f) Combined potential emission sensitivities (PESs) for 10-day FLEXPART back-trajectories launched from the study site for the duration of the campaign (30/05/2017–01/07/2017) for 0–2500 m above ground level (AGL), g) FLEXPART output map (0–2500 m AGL) and average position (m above sea level) for a 10-day back-trajectory launched from the study site (orange star) on 15/06/2017 15:00 Z as a representative example of the path taken during high pressure, clear weather dominated by katabatic winds from the ice sheet center, and h) FLEXPART output map (0–2500 m AGL) and average position (m above sea level) for a 10-day back-trajectory launched from the study site on 20/06/2017 09:00 Z as a representative example of the path taken during a period of inclement weather that delivered snow to the site. In panel c, gray boxes indicate when OPC measurements in panel (a) were being made; snow symbols indicate the timing of sampled snowfall events (June 17 and 20); vortex symbols indicate Coriolis μ air sampler collection time points (corresponding to Figure 2c); + and × indicate launch time points for FLEXPART simulations in panels (g) and (h). See Figures S5, S7, and S8 for corresponding 2016 results.

Combining the 2017 fresh snow particulate mass load and estimated snowfall rates (Figure S6, Table S10) yielded an estimated rate of particulate delivery via snowfall of 880 ± 240 mg·m^–2^·year^–1^ (Figure 1). This is within the same order of magnitude as dust flux estimates from deep ice cores from central North Greenland NGRIP (0–400 mg·m^–2^·year^–1^)^50,51^ and East Greenland Renland (680 mg·m^–2^·year^–1^).^39^ Note that dust deposited via rainfall was not measured in the present study, and thus, the total wet deposition value (i.e., all precipitation) is likely higher than what is reported. Multiyear, seasonal measurements of dust deposition via snow and rain would therefore provide valuable insight into the total dust flux to the surface of the GrIS. During the 2017 campaign, snowfall corresponded to periods of low air pressure and relative humidity >90% on days with cloud cover and stagnant or westerly winds (Figure 5c–e). These occasional low-pressure systems from the coast interrupted the dominant weather at the site, which consisted of stable katabatic winds from the southeast (142 ± 43.1°) at a mean speed of 4.7 ± 1.9 m·s^–1^ (Figure 5e). These winds are driven by the shape of the GrIS^52^ and are typically accompanied by periods of clear, precipitation-free weather. In 2017, the mean air pressure, air temperature, and relative humidity were 892 ± 4 hPa, 0 ± 2.1 °C, and 76.8 ± 6.0%, respectively (Figure 5c,d). Compared to 2017, the weather during the 2016 campaign was more stable, with more sunny, cloudless days and consistent katabatic winds (see SI text and Figure S8 for details).

### Linking Local Aerosol Delivery to Large-Scale Atmospheric Processes

The FLEXPART model PES outputs (Figure 5f–h, S8) revealed that in the 10 days preceding particle release, airmasses that arrived at the study site traveled long distances, reaching as far back as Northern Canada, Northern Europe, and the mid-Atlantic. Most of this long-range transport occurred at high altitudes (e.g., >2500 m AMSL), and aggregating the PES outputs for all simulations for the duration of the 2016 and 2017 campaigns shows the highest PES values over Greenland (Figure 5f, S8e). This result suggests that the study site is more sensitive to particle emissions from Greenlandic locations than locations further away. Movies of PES outputs are listed in the SI file and are available as a linked data deposition. Airmasses were typically only in close proximity to the surface for brief periods immediately before (<24 h) reaching the site (Figure 5g,h lower panels). More specifically, during periods of clear, sunny weather dominated by katabatic winds, PES shows airmass circulation predominantly over the GrIS before descending near the center of the ice sheet and traveling from the southeast to the study site (Figure 5g, S8f). During such periods, the mean airmass transport path is at a high altitude (Figure 5g, lower panel) with little interaction with the boundary layer before it descends near the middle of the ice sheet. In these instances, any aerosols from the free troposphere would likely be deposited in the center of the ice sheet,^53^ and thus, the airmass would be depleted of aerosols by the time it arrived at our study site. Such deposition of dust from high altitude transport in the center of the ice sheet may explain why dust in ice cores from central GrIS locations has REE signatures that indicate a contribution from East Asian sources.^39^ In contrast, during periods of inclement weather that delivered snow to the site, PES shows pronounced transport from the west coast of Greenland, with the average transport path interacting with the boundary layer for longer prior to reaching the study site (Figure 5h lower panel, S8g). These boundary layer interactions would have occurred above the nearby proglacial plains, thereby providing opportunities for the air to collect local mineral dust. This finding is consistent with the REE signatures measured for the mineral dust deposited in snow that indicated local Greenlandic dust sources (Figure S4a,b). The largest proximal dust emission source is the forefield region near Kangerlussuaq, west of the site (Figure 1).^14,54^ The Kangerlussuaq area experiences more dust events in spring and autumn than in summer and winter,^14^ indicating that dust emission rates experience seasonal variability in addition to interannual variability. Increased dust emissions have already been documented in coastal Greenland locations as a result of reduced snow cover,^55^ and dust events will likely become more frequent with continued climate warming and glacial retreat. With respect to wet deposition, our study site receives the most snowfall in the months of September and October (Figure S6), and since autumn is a time of more frequent dust events near Kangerlussuaq,^14^ it is likely that autumn is a season of high mineral dust deposition rates via snowfall. No winter airborne dust concentration measurements exist for our study site, but it is expected that there is less dust transported from the Kangerlussuaq region during winter once the proglacial plains are snow-covered.

### Phosphorus Deposition and Glacier Ice Algal Bloom Development

Having characterized the mineral dust delivered *on-ice* via dry deposition and snowfall, as well as concentrations of dissolved solutes in snow at the site, we estimated the potential for these constituents to provide phosphorus to the ice surface for glacier ice algal bloom development. Combining the mean concentration of dissolved phosphorus in fresh snow (1.34 μg·L^–1^, *n* = 2) and the mean estimated snowfall rate (299 kg·m^–2^·year^–1^, see SI for details), we calculated the rate of dissolved phosphorus deposition as 0.4 mg·m^–2^·year^–1^ (Table 1). Using the hydroxylapatite abundance of 0.3 wt % and the mineral dust delivery rates for snow (2017: 880 mg·m^–2^·year^–1^) and dry deposition (mean of 2016 and 2017: 508 mg·m^–2^·year^–1^) generated phosphorus delivery rates of 0.5 mg·m^–2^·year^–1^ in mineral dust delivered by snow and 0.3 mg·m^–2^·year^–1^ for phosphorus in mineral dust delivered via dry deposition (Table 1). Note that this hydroxylapatite abundance of 0.3 wt % is from one snowfall event in 2017, and better constraining the mineralogy of dust in fresh snow falling on the GrIS would certainly be of value. Despite this, the measured value is very similar to the average hydroxylapatite abundances here determined for weathered snow (0.4 wt %, *n* = 2) and for high algal biomass ice and dispersed cryoconite ice (0.4 wt %, *n* = 5) collected in the same seasons at the same study location.^16^

**Table 1:** Estimated Phosphorus Deposition Rate via Snowfall and Dry Deposition, Corresponding Estimated Glacier Ice Algal Cell Abundances, and Carbon and Nitrogen Biomass Accumulation Rates^a^,^b^

	Dissolved *P*_(snow)_	Mineral *P*_(snow dust)_	Mineral *P*_(dry deposition)_	Total *P*
P deposition (mg·m^–2^·year^–1^)	0.4 (0.08)	0.5	0.3	1.2
Glacier ice algal abundance, 1 m ablation (cells·mL^–1^)	2.9 × 10^3^ (5.7 × 10^2^)	3.6 × 10^3^	2.1 × 10^3^	8.6 × 10^3^
Carbon accumulation (kgC· m^–2^·year^–1^)	303 (61)	379	227	909
Nitrogen accumulation (kgN· m^–2^·year^–1^)	13 (2.6)	16	10	39

a Assuming 1 M of seasonal ablation and 100% efficiency of phosphorus use by glacier ice algae; values in parentheses for dissolved phosphorus indicate the values corresponding to 80% solute loss by elution during melting; the value calculated for phosphorus delivered via dry deposition is calculated assuming that this dust has the same mineralogical composition as that delivered in snow.

b Calculated using cellular nutrient values: *C* = 106 pg·cell^–1^, *N* = 4.5 pg·cell^–1^, *P* = 0.14 pg·cell^–1^ (from ref 56).

Combining these calculated rates with the glacier ice algae cellular *C*:*N*:*P* ratio reported by Williamson et al.,^56^ we estimated the glacier ice algal bloom potential for each phosphorus delivery pathway. If an ablation rate of 1 m·year^–1^ is applied, the dissolved phosphorus delivered by snow can fuel the growth of glacier ice algae in abundances of 2.9 × 10^3^ cells·mL^–1^, which upscales to algal biomass production in the amount of 303 kgC·km^–2^·year^–1^ (Table 1). This large potential biomass yield is a product of the low macronutrient requirements (and therefore high cellular *C*:*N* and *C*:*P* ratios) of glacier ice algae^56,57^ such that even a small amount of phosphorus delivered to the ice can generate a lot of biomass. It should be noted that this estimate assumes 100% of the delivered phosphorus is used to fuel glacier ice algae growth and that while our measurements of solute concentrations did not indicate notable elution, as much as 80% of solutes in snow are estimated to be eluted during the earliest stages of melting.^58^ If the dissolved phosphorus in snow is eluted prior to microbial uptake, then the resulting glacier ice algal cell densities and corresponding biomass production yields decrease accordingly (Table 1). If phosphorus delivered in mineral dust via snowfall and dry deposition is also considered, these contributions can support additional algal growth in abundances of 3.6 × 10^3^ and 2.1 × 10^3^ cells·mL^–1^, respectively, yielding a total glacier ice algal cell density of 8.6 × 10^3^ cells·mL^–1^ (Table 1). This cell density is within the range (typically <10^5^ cells·mL^–1^) reported by numerous studies on the GrIS.^31,32,56,59^ The relationship between ice algal densities and albedo is well-known,^5,20,31,32,60,61^ and the calculated cell density translates to a broadband albedo value of ∼0.3–0.4. Such a value would yield substantial surface melting and is consistent with the melting we documented in the 2016 season.^20^

Given the regional extent of glacier ice algal blooms in the Dark Zone of the GrIS,^20^ the impact of atmospherically delivered phosphorus fueling algal growth likely extends beyond our study location. The algal biomass supported by the phosphorus delivered via mineral dust translates to a calculated additional 379 kgC·km^–2^·year^–1^ (dust in snow) and 227 kgC·km^–2^·year^–1^ (dry deposited dust), generating a total biomass accumulation of 909 kgC·km^–2^·year^–1^ (Table 1). Using the cellular C/N ratio for glacier ice algae from Williamson et al.,^56^ this equates to 39 kgN·km^–2^·year^–1^. These values represent an upper threshold on algal biomass accumulation, as 100% utilization by glacier ice algae is unlikely. The incorporation of mineral phosphorus into biomass would not be instantaneous and would be dependent on mineral weathering rates, potentially facilitated by the activity of the bacteria and fungi documented in the microbial community (Figure 4). Bacterial and fungal communities have been shown to accelerate the release of phosphate from apatite by 2 orders of magnitude compared to abiotic control experiments through the release of organic acids, thereby providing a method for the microbial community to access mineral-hosted phosphorus.^62^ Dissolution of mineral dust in snow has been credited with causing seasonal increases in dissolved phosphate in other cryosphere environments, including melting snowpack on the Foxfonna ice cap in Svalbard.^63^ Mineral dust that accumulates on the ice sheet surface may act as an *on-ice* phosphorus reservoir for the ice sheet microbial community, gradually dissolving and releasing phosphorus over time. Once liberated, at least some of the dissolved nutrients are retained in the glacier ice algal habitat,^43^ and phosphorus weathered from mineral grains is likely recycled within the microbial community. Snowfall over southwest Greenland is projected to increase^64^ and thus may deliver more mineral and dissolved phosphorus to the ice surface in the future, while similarly projected increased rainfall and surface runoff may accelerate the removal of dust and associated nutrients. Additionally, the microbial community data presented for the air samples (Figure 4) indicate that glacier ice and snow algae may be transported, at least locally, by wind, which may provide a means of inoculating fresh ice and snow surfaces with these organisms. This process may become important in a warming climate as locations further north and further inland become more suitable for the growth of these cryophilic algae. Collectively, these results indicate that biogeochemical links between atmospherically delivered nutrients and glacier ice algal bloom development and transport may have important implications for surface ice melting in this region of the GrIS in a changing climate.

## Supplementary Material



## Data Availability

Aerosol measurement data sets (10.5281/zenodo.17985533) and FLEXPART PES outputs (10.5281/zenodo.17990403) are deposited on Zenodo, and the microbial community sequencing data are deposited on NCBI under BioProject PRJNA1287549.

## Notes

Methods related to optical particle counter measurements, electron microscopy, mineralogy, meltwater chemistry, microbial community composition, dust deposition velocity and flux calculations, and meteorology; results including the 2016 particulate size measurements, mass fluxes, meteorological data, and FLEXPART outputs; modeled snowfall data; 2017 solar radiation measurements; dust REE data; aerosol sample and data summary tables including particulate size measurements, dust concentrations, mineralogy, REE measurements, and additional EDS measurements; meltwater chemistry and dissolved phosphorus measurement result comparison; captions for supplemental movies and data sets associated with this article (PDF)
