# Supplementary material for: Atmospheric Deposition of Local Mineral Dust Delivers Phosphorus to the Greenland Ice Sheet
Source: Environ Sci Technol. 2026 Jan 13;60(3):2515–27. doi: 10.1021/acs.est.5c13873 (PMC12854737; doi:10.1021/acs.est.5c13873)
Supplement: Supplementary file 1 [file es5c13873_si_001.pdf]

Supporting Information for:

Atmospheric deposition of local mineral dust delivers phosphorus to the Greenland Ice Sheet

Jenine McCutcheon[1,2*], James B McQuaid[1], Nuno Canha[1,3], Sarah L Barr[1], Stefanie Lutz[4,5], Vladimir Roddatis[4], Sathish Mayanna[4,6], Andrew J Tedstone[7,8,9], Martyn Tranter[7,10], and Liane G Benning[1,4,11]

*Email: jenine.mccutcheon@uwaterloo.ca

This file includes:

Supporting methods and results text
Figures S1 to S8
Tables S1 to S10
Captions for Movies S1 to S14
Captions for Datasets S1 to S7
SI References

Supporting Information Text

Methods

Optical particle counter

The OPC used in this study is rated for use in temperature and relative humidity ranges of -20 to +50°C and 0 to 95%, respectively, and for particle count rates of up to 10,000 particles per second (1). The power limitations of the location necessitated using an OPC with low power requirements, and tests comparing this OPC to reference instruments yielded R^2 values of 0.94–0.99 and size-dependent particle detection efficiencies of 78–101% (2). The raw OPC data consisted of particle counts in 16 size bins covering the size range of 0.38 – 17 µm. These data were re-binned into five size bins: < 1 µm, 1 – 2.1 µm, 2.1 – 5 µm, 5 – 10 µm, and > 10 µm and were used to generate 10 minute averages. Time periods in the sampling windows for which no data are presented represent one of the following: 1) the OPC was powered off due to insufficient solar power; 2) the OPC was powered off due to activity in camp that could contaminate the measurements (e.g., helicopter activity); or 3) the Alphasense OPC-N2 has been reported to overestimate particle counts in very high relative humidity conditions (3), thus producing unrealistically high counts. These steps were taken to optimize the quality of the data used in the subsequent calculations.

Electron microscopy

Elemental mapping using energy dispersive spectroscopy (EDS) was conducted using a ThermoFisher Scientific EDS detector attached to a Zeiss FE SEM operated at 20 kV and working distance of 10 mm, with additional corresponding imaging conducted at 15 kV. The presented EDS results from the June 28, 2017 (GrIS-17-Cr-16) Coriolis® air sample represent n=7 regions analyzed, with a summed surface area of 7.1 mm^2. EDS was also conducted on two aerosol filters (GrIS-17-Aero-1 and GrIS-17-Aero-4) from 2017 (Table S3). Particle densities on these filters were lower than on GrIS-17-CR-16 and thus the data from the multiple regions in GrIS-17-Cr-16 are presented in the main manuscript. In all EDS data, C has been excluded from the elemental summaries due to the presence of C in the underlying polycarbonate filter. Additional secondary electron (SE)-SEM of the aerosol, Coriolis®, and snow particulate samples was conducted using a Hitachi 8230 SEM operated at 2 kV and working distance of 4 mm at the Leeds Electron

Microscopy and Spectroscopy Centre.

Snow dust mineralogy

The 1996 ICDD and 2016 COD databases were used to complete phase identification using DIFFRAC*plus* Eva v.2 software (4). Topas V 4.2 (4) and the fundamental parameters approach (5) were used to conduct the Rietveld refinements (6-8). The XRD patterns exhibit the effects of non-ideal particle size statistics and preferred orientation on some phases due to the reliance on hand grinding, which can result in higher R_{wp} values. Multiple K-feldspar, plagioclase feldspar, and orthopyroxene structures were used in a single refinement because this approach improved both the visual fit to the observed XRD patterns and the fit statistics. This may indicate that the dust in the snow samples was derived from multiple source rocks of differing composition, which is reasonable considering the source of the sample. Mineral phases identified were then grouped into the following classes: quartz, plagioclase feldspars (albite/andesine/anorthite), amphiboles (refined using the structure of actinolite), potassium feldspars (orthoclase/microcline), pyroxene (enstatite/augite/diopside), and micas (refined using the structure of muscovite).

Meltwater chemistry

For the ICP-MS measurements, the limit of detection (LOD), limit of quantification (LOQ), and uncertainty (%) are reported for each element in Table S7. For phosphorus, the LOD, LOQ, and uncertainty were 0.17 $\mu g \cdot L^{-1}$, 0.57 $\mu g \cdot L^{-1}$, and 4.81%, respectively. Additional dissolved phosphorus measurements were made using a 100 cm WPI Liquid Waveguide Capillary Cell (LWCC) in conjunction with an Ocean Optics USB2000 + spectrophotometer with a precision of 5.1% and a LOQ of 0.37 $\mu g \cdot L^{-1}$. These additional measurements were made for comparison purposes (Table S8).

Microbial community composition

DNA was extracted from all samples using the PowerWater DNA Isolation kits (MoBio Laboratories). The 16S rRNA and18S rRNA amplicons were prepared following the Illumina "16 S Metagenomic Sequencing Library Preparation" guide, prior to sequencing on the Illumina MiSeq using paired 300-bp reads at the University of Bristol Genomics Facility, Bristol, UK following the same steps as outlined in McCutcheon*, et al.* (9). The sequenced 16S and 18S rRNA gene libraries were individually imported into QIIME2 (v2024.10) (10). Quality filtering was performed using the DADA2 plugin (parameters: --p-trunc-len-f=280, --p-trunc-len-r=200, --p-trim-left-f=10, and --p-trim-left-r=10) (11). Amplicon sequence variants (ASVs) were inferred and

subsequently clustered into operational taxonomic units (OTUs) at 99% sequence similarity. Taxonomic classification was conducted using the classify-sklearn method against the SILVA reference database (release 138) (12-14).

The resulting feature tables were imported into R (v4.4.3) for downstream analysis (15). OTU tables, taxonomic assignments, and sample metadata were combined into phyloseq objects using the phyloseq package (v1.36.0) (16). OTUs with zero counts across all samples were removed, as well as those taxonomically annotated as "Chloroplast," to reduce potential bias. All samples were then rarefied to even sequencing depth based on the sample with the lowest read count (16S: 7,160 reads; 18S: 5,471 reads) using the rarefy_even_depth function. Rarefaction curves were generated with the ggrare function in the from the phyloseq.extended package (v0.1.4.1) to assess whether sequencing depth was sufficient across samples. Bar plots visualizing taxonomic composition were created using ggplot2 (v3.5.1) (17).

Dry deposition velocity and flux calculations

Code related to the following calculations is available here: https://github.com/orgs/Black-and-Bloom-Project/repositories. The deposition velocity for a reference height z_{ref} is defined as the constant of proportionality between the concentration at the reference height and the downward flux T, as defined by Equation 1:

$$v_{ds} = v_{ds}(Z_{ref}, \delta, \chi) = v_g \left(\frac{\delta}{Z_{ref}}\right)^{-\frac{v_g}{\chi}} \qquad \text{[Equation 1]}$$

Where v_{ds} is the deposition velocity (m·s^{-1}), Z_{ref} is the reference height (2 m), δ is the deposition height (m), χ is the parameter that includes information about wind velocity, and v_g is the particle gravitational settling velocity (m·s^{-1}) that is defined by Equation 2:

$$v_g = \frac{g \times C_c \times \rho_p \times {d_p}^2}{18 \times v_a} \qquad \text{[Equation 2]}$$

Where g is the acceleration of particle due to gravity (9.81 m·s^{-2}), C_c is the Cunningham slip correction factor (dimensionless), ρ_p is the particle density, d_p is the physical diameter of the particle (m) and v_a is the kinematic viscosity of air (m^2·s). A particle density of 2.5 g·cm^{-3} was used (18). The kinematic viscosity of air (m^2·s) for air temperatures between -173 and 227 ºC can be calculated as a function of air temperature (in ºC), following Equation 3 defined Andreas (19):

$$v_a(T) = 1.326 \times 10^{-5}(1 + 6.542 \times 10^{-3}T + 8.301 \times 10^{-6}T^2 - 4.840 \times 10^{-9}T^3)$$

[Equation 3]

The Cunningham slip correction factor varies with particle diameter and, for particles below 1 μm, it is necessary to apply the correction defined by Equation 4 (20):

$$C_c = 1 + \frac{6.21 \times 10^{-4} T}{d_p} \quad \text{[Equation 4]}$$

Where d_p is the particle diameter (in μm) and T is the air temperature (in K). The χ parameter of Equation 1 is defined by Equation 5 (21):

$$\chi = \beta \times k \times u^* \quad \text{[Equation 5]}$$

Where β is a factor that accounts for the difference between the diffusivity of momentum and a scalar contaminant (22), which for aerosols and for stable conditions its value is assumed to be 1, k is the *von* Karman constant (value of 0.4) and u^* is the friction velocity obtained from the wind velocity ($m \cdot s^{-1}$). The friction velocity may be calculated from the neutral surface wind speed (U_{10N}, in $m \cdot s^{-1}$) following the second-order polynomial equation defined by Zou, *et al.* (23), according to Equation 6 for onshore wind:

$$u_* = \begin{cases} 0.0015\, {U_{10N}}^2 + 0.0099 U_{10N} + 0.062, & for\ onshore\ wind \\ 0.0012\, {U_{10N}}^2 + 0.016 U_{10N} + 0.052, & for\ offshore\ wind \end{cases} \quad \text{[Equation 6]}$$

The mass concentration of particles (in $\mu g \cdot m^{-3}$) was obtained from the OPC particle counts, using the Equation 7 to calculate the aerosol mass loading (PM) (24) considering the particles as spheres, and, afterwards, dividing by the flow rate (2.2×10^{-4} $m^3 \cdot min$).

$$PM = \rho \sum_i N_i \frac{\pi}{6} d_{p,i}^3 \quad \text{[Equation 7]}$$

Where N_i is the number concentration for a given size bin, $d_{p,i}$ is the mean diameter for a given size bin, and ρ is the particle density. The dry deposition flux of particles is defined in Equation 8 (25, 26), and, therefore, for an individual interval i and for a particle of a specific diameter d, the dry deposition flux of particles was calculated based on Equation 9.

$$\text{Flux} = \text{concentration} \times \text{deposition velocity} \quad \text{[Equation 8]}$$

$$\text{Flux}_\text{i} = \text{Concentration}_\text{i} \times \text{v}_\text{d,i} \quad \text{[Equation 9]}$$

Meteorology

The accuracy of the meteorology measurements made at the study site are: temperature (± 0.2°C), relative humidity (± 2% at 5 – 95 % RH, ±2.5% <5% and >95% RH), incident solar radiation (±5%), wind speed (± 0.1 $m \cdot s^{-1}$, ±1.1% of reading), and wind direction (±4°).

Results

Dissolved phosphorus concentration measurement comparison

A comparison of the phosphorus concentrations measured using ICP-MS and LWCC methods are made in Table S8. The ICP-MS measurement values are typically slightly higher than the LWCC values. This may be due to a small amount of dissolved organic phosphorus (DOP) and/or phosphorus-bearing colloidal material being captured in the ICP-MS values.

Snowfall rate estimation

The mean annual snowfall at the site for the period of 1980 – 2019 as estimated by MAR v3.11.2 was 299 ± 81 $kg \cdot m^{-2} \cdot year^{-1}$ (Fig. S6, Table S10), within the 120 – 500 $kg \cdot m^{-2} \cdot year^{-1}$ range of snowfall measured from ice cores throughout Greenland (27). Our study years, 2016 and 2017, experienced typical snowfall rates, with estimates of 220 and 284 $kg \cdot m^{-2} \cdot year^{-1}$, respectively, however, the lack of fresh snowfall during the 2016 campaign dates prevented an estimation of the total dust delivery for that year.

Meteorology results

Measured incident solar radiation provides an indication of cloud cover, and the mean incident solar radiation during the 2017 campaign was 330.8 ± 262.8 $W \cdot m^{-2}$. Cloudless days exhibited a clear diurnal trend and midday maximums of ~750 $W \cdot m^{-2}$, and scattering on overcast days caused the diurnal trend to be less distinct, with maximum values exceeding 1200 $W \cdot m^{-2}$ (Fig. S7). The measured mean meteorological conditions in 2016 were: air pressure (895.7 ± 6.3 hPa), air temperature (0.8 ± 1.6°C), relative humidity (91.5 ±7.7 %), wind direction (127.9 ± 33.0°), wind speed (3.1 ± 1.5 $m \cdot s^{-1}$), and incident solar radiation (267.8 ± 251.7 $W \cdot m^{-2}$) (Fig. S8a-d).

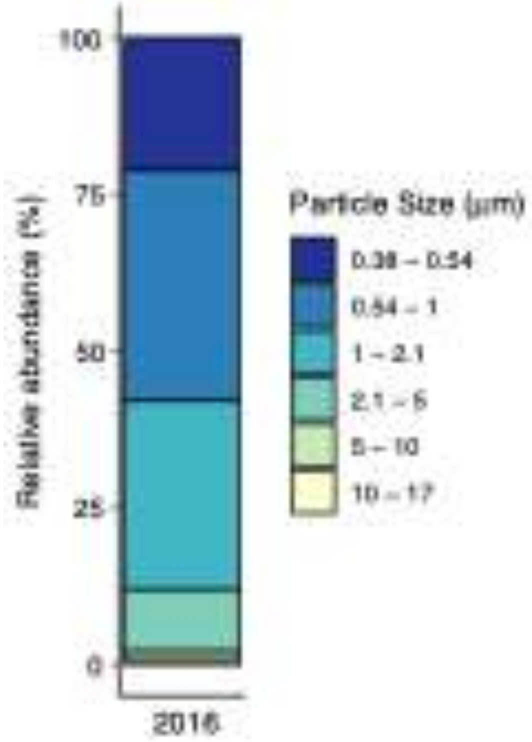

Fig. S1. Size distribution of airborne particulate matter collected onto filters in 2016, measured using SEM (filters; n=7; particles: n= 4,466).

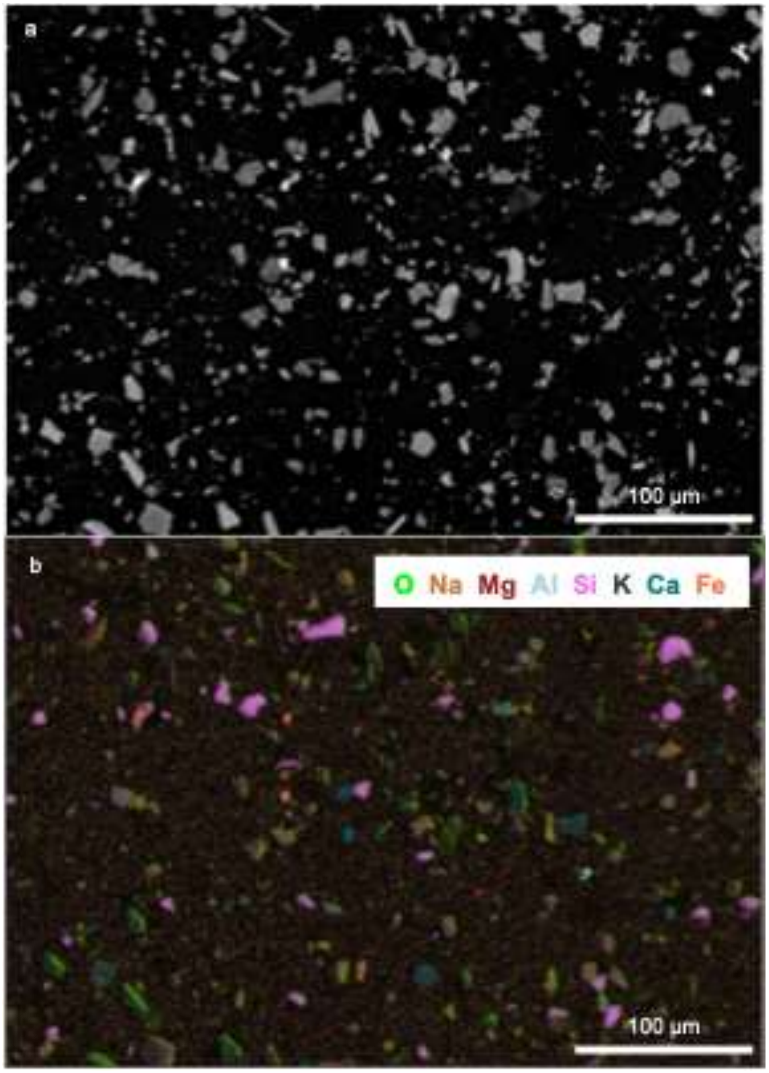

Fig. S2. a) BSE-SEM micrograph and corresponding b) elemental map produced using EDS for sample 17-CR-16, which consisted of mineral dust on a polycarbonate filter collected using the Coriolis® in 2017. This is one of the seven large regions used to produce the mean dust elemental composition presented in Figure 2d of the main text.

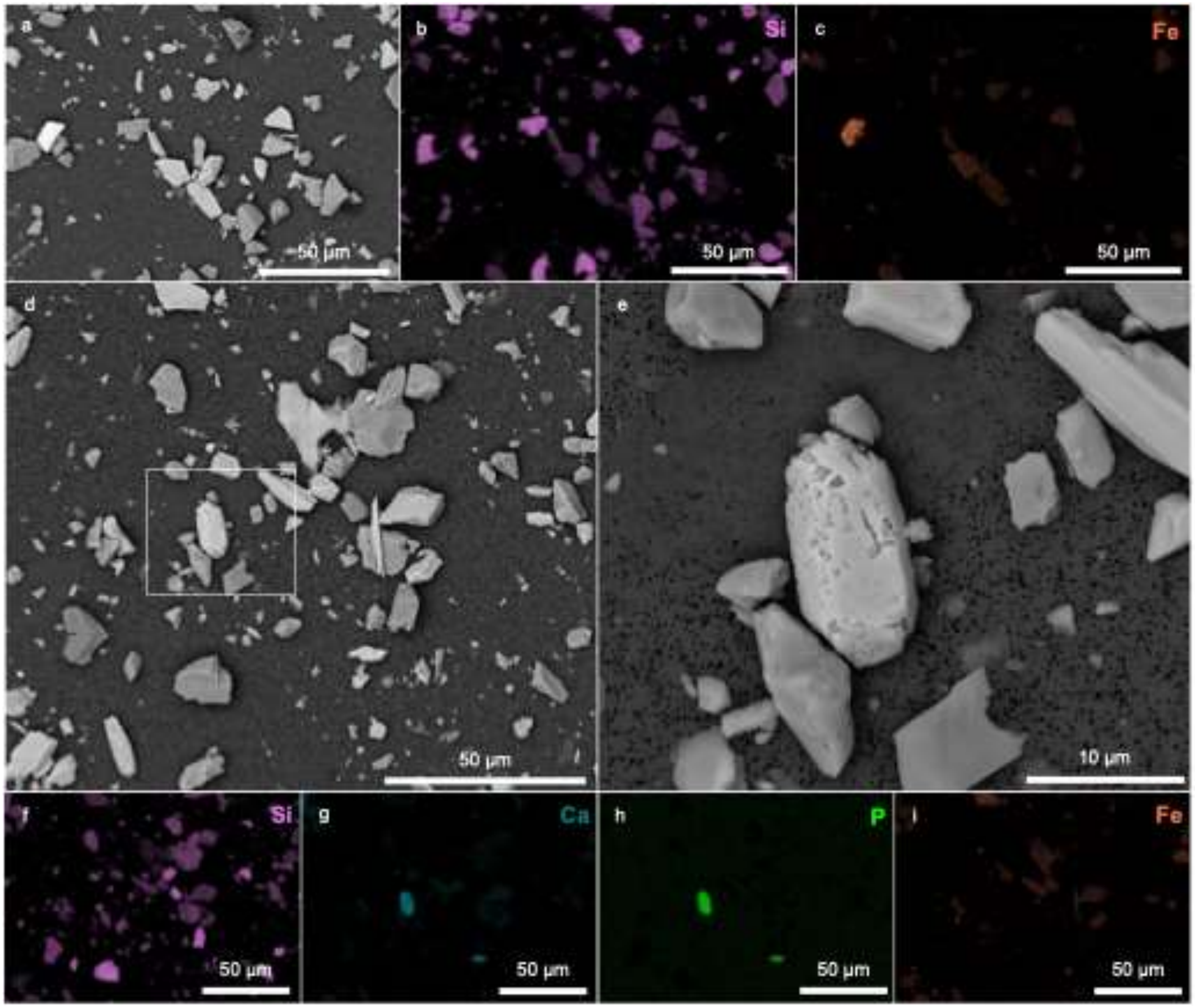

Fig. S3. a) BSE-SEM micrograph, b) Si EDS map, and c) Fe EDS map corresponding to the micrograph and P and Ca maps displayed in Figure 2e–g in the main text. d) Low magnification and e) high magnification BSE-SEM micrographs showing the presence of another Ca-P grain. Region shown in e) indicated by box in d). Elemental maps generated using EDS showing the distribution of f) Si, g) Ca, h) P, and i) Fe for the region shown in d), showing two grains presumed to be apatite based on the collocated Ca and P. Sample: GrIS-17-CR-16; mineral dust on a polycarbonate filter collected using the Coriolis® in 2017.

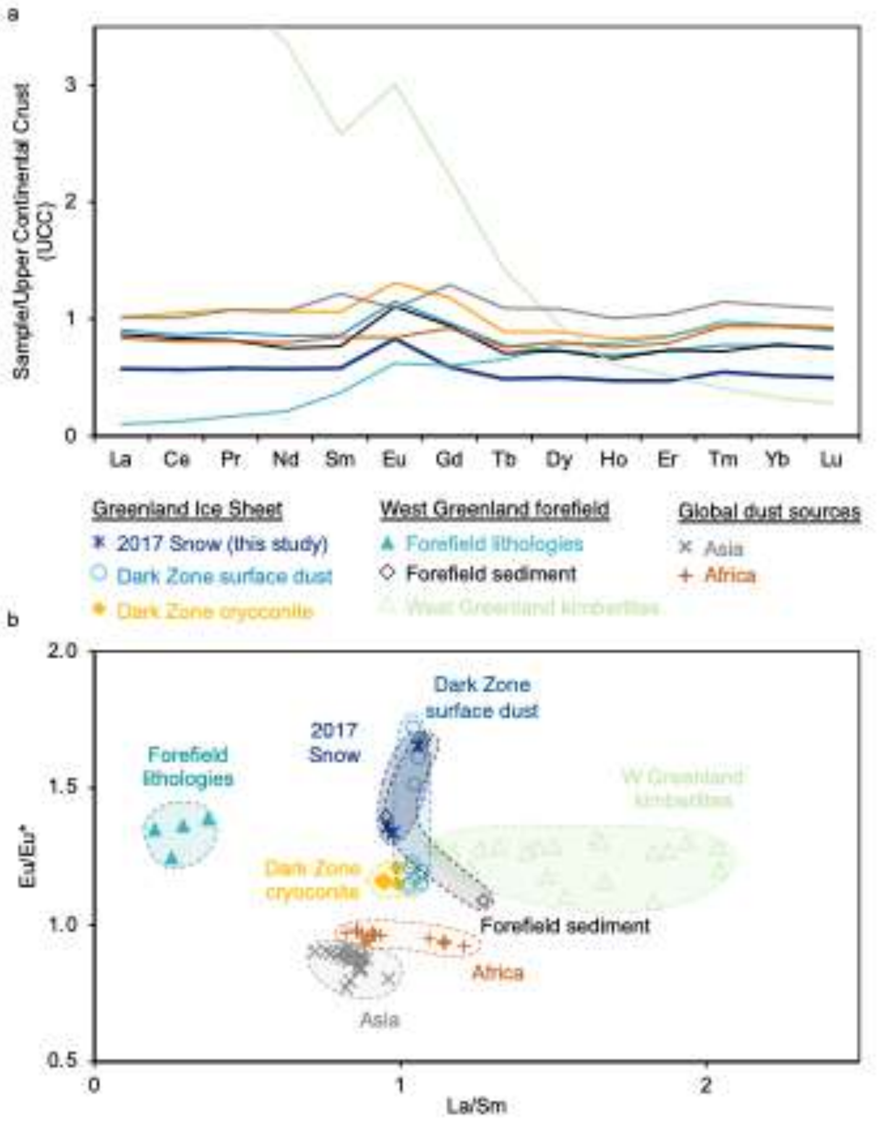

Fig. S4. a-b) Rare earth element (REE) measurements normalized to the upper continental crust (UCC) for mineral dust in snow (this study, n=3) in comparison to surface dust at the site and forefield lithologies (9), dust in nearby cryoconite holes (28), forefield sediments (29), and suite of kimberlite samples from West Greenland (30), and characteristic dust from Asia (31), and Africa (32). REE concentrations in Table S6. The REE plots, with the addition of the new data for dust in snow, are replotted from McCutcheon*, et al.* (9) as permitted under the Creative Commons Attribution 4.0 International License (https://creativecommons.org/licenses/by/4.0/) for that article, published in Nature Communications in 2021.

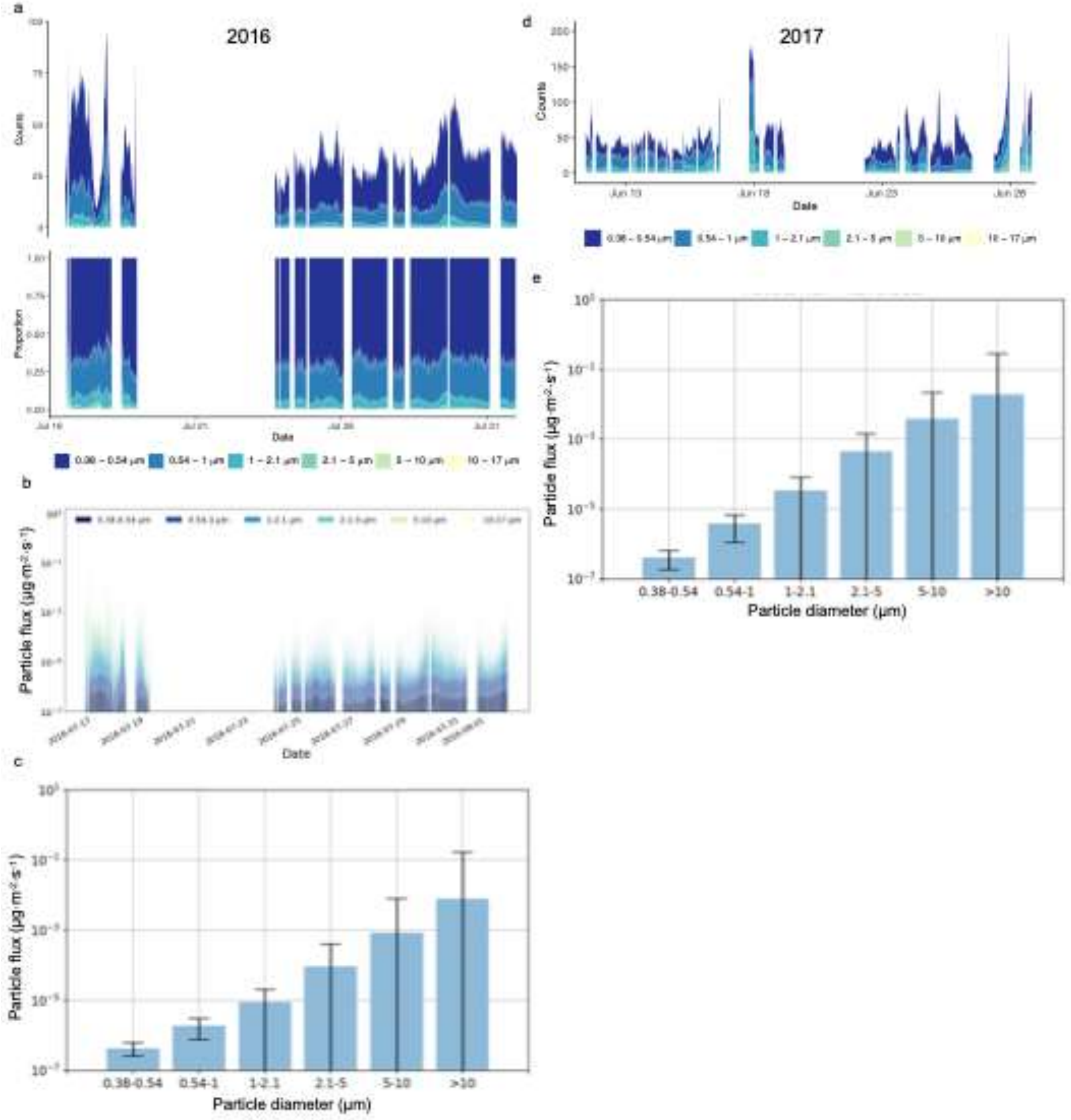

Fig. S5. 2016 campaign measurements of a) aerosol particle counts and proportion by size and b) mass distribution by size bin measured using an optical particle counter, and c) particle mass flux by size (binned to match OPC bins). 2017 measurements of d) OPC aerosol particle counts (matching Fig. 5a in main text), and e) particle mass flux by size.

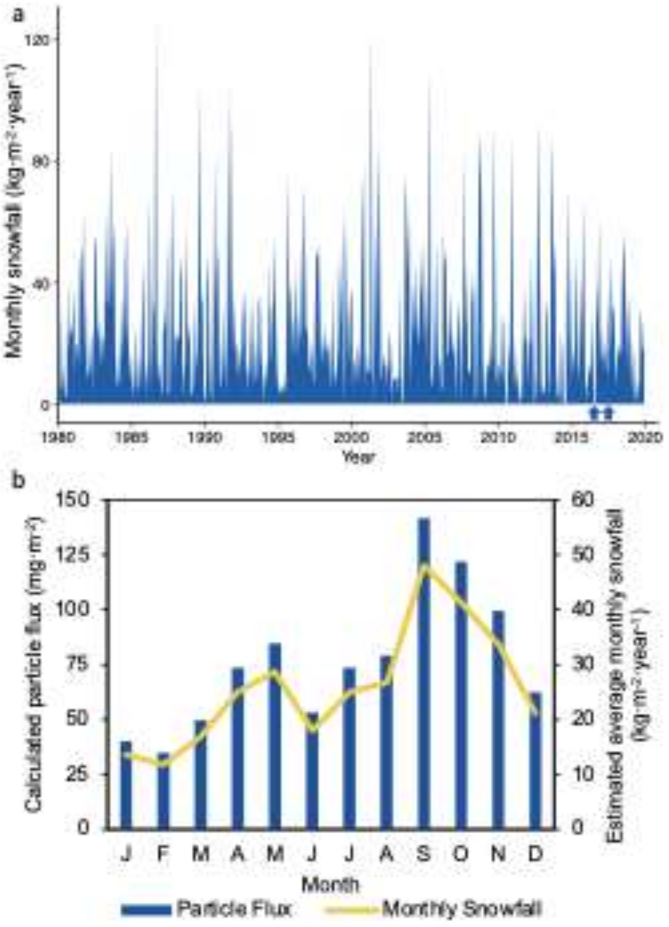

Fig. S6. a) Modelled monthly snowfall (kg·m^{-2}·year^{-1}) from MAR v3.11.2 forced by ERA5 reanalysis since 1980, with blue arrows on the right end of the X-axis indicating our sampling years of 2016 and 2017. b) Mean monthly snowfall (kg·m^{-2}·year^{-1}) was calculated using the 40 years of modelled snowfall data (1980 – 2019) and corresponding calculated particulate mass flux (mg·m^{-2}) using the particulate mass load from our fresh 2017 snow, expressed by calendar month.

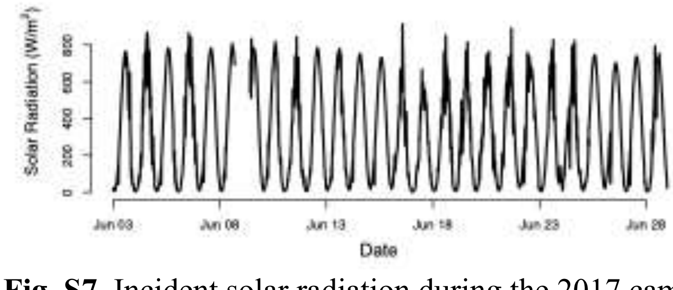

Fig. S7. Incident solar radiation during the 2017 campaign.

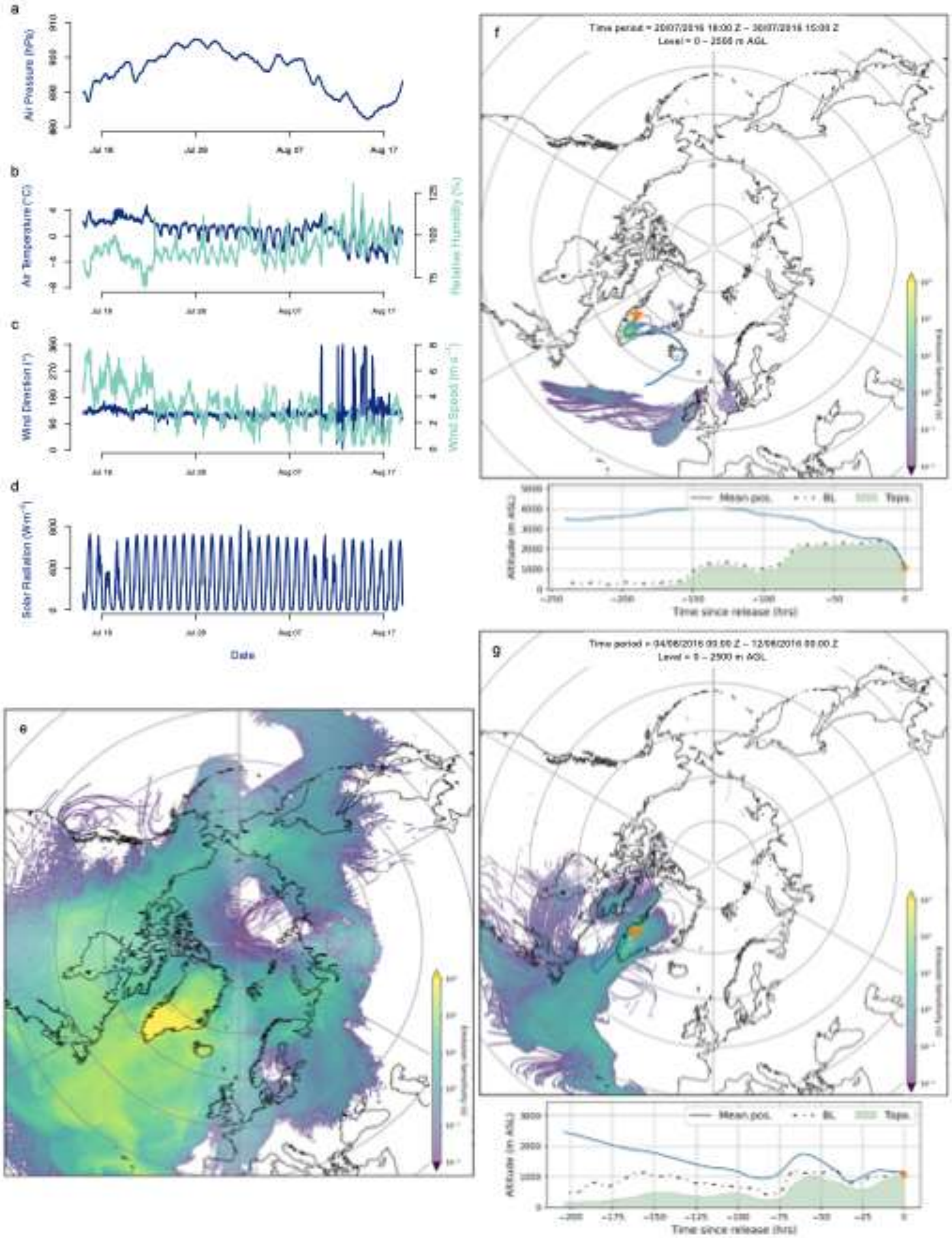

Fig. S8. Time resolved measurements from the S6 automatic weather station for the duration of the 2016 campaign of a) air pressure, b) air temperature and relative humidity, c) wind speed and direction and d) incident solar radiation; e) combined FLEXPART output map for 10-day back-trajectories launched from the campaign site for the duration of the campaign (10/07/20216 – 22/08/2016) for 0 – 2500 m above ground level (AGL), f) FLEXPART output map (0 – 2500 m AGL) and mean path (m above sea level) for a 10-day back-trajectory launched from the campaign site on 30/07/2016 15:00 Z as an example of the modelled aerosol transport path during clear

weather dominated by katabatic winds, and f) FLEXPART output map (0 – 2500 m AGL) and mean path (m above sea level) for a 10-day back-trajectory launched from the campaign site on 12/08/2016 00:00 Z as an example of the path taken during a period of inclement weather at the site. Note for b: the relative humidity values exceeding 100% are due to being measured with respect to water by the weather station; these values have not been recalculated with respect to ice for temperatures below freezing (33).

Table S1. Summary of analyses conducted in this study and corresponding air, snow, and ice sample or measurement numbers.

**Measurement type**	**2016 sample numbers**	**2017 sample numbers**
OPC aerosol size	Time-resolved; n=10,791 particles measured	Time-resolved; n=14,385 particles measured
SEM particle size collected on air filters	n=4,466 particles measured, collected onto 7 different filters from >89,000 L of air over >197h of sampling	n=14,070 measured, collected onto different 4 filters from >64,000 L of air over >57h of sampling
SEM-EDS of mineral dust collected on air filters	Insufficient particle mass to measure	14 regions from 3 samples
Air 16S and 18S amplicon sequencing	3 samples; >30,000 L of air per sample	7 samples; >6,000 L of air per sample
Snow 16S and 18S amplicon sequencing	No snowfall during campaign	4 samples
Fresh snow particle mass loading	No snowfall during campaign	2 (1 from each snow event)
Fresh snow mineralogy and REE	No snowfall during campaign	1
Fresh snow meltwater ICP-MS	No snowfall during campaign	2 (1 from each snow event)
Weathered snow particle mass loading	No snow to sample	8
Weathered snow mineralogy and REE	No snow to sample	2
Weathered snow meltwater ICP-MS	No snow to sample	5
Surface ice*	0	1
Clean core ice	0	1
Meteorological data	S6 automatic weather station data used (Utrecht University Institute for Marine and Atmospheric Research); hourly data >1,100 measurements per parameter	>36,000 measurements per parameter

*additional surface ice data is available in McCutheon et al. 2021.

Table S2. Sample details for aerosol particulate matter collected via active sampling either onto polycarbonate filters mounted 2 m above the ice surface or into ultra-high quality water using the Bertin Instruments Coriolis ® µ air sampler. Coriolis samples were typically collected in pairs for deposition onto DNA and polycarbonate filters, with equal sample collection durations and volumes for each filter type.

**Sample ID**	**Collection Dates**	**Collection method**	**Sampling duration (hh:mm)**	**Air volume (L)**	**Particles measured**	**Mean particle diameter (µm)**	**Particle diameter standard deviation (µm)**
GrIS-16-Aero-2	19-07-2016 – 21-07-2016	Active sampling onto PC filter	38:40	16240	303	1.16	0.68

GrIS-16-Aero-8	23-07-2016 – 24-07-2016	Active sampling onto PC filter	22:30	9450	376	0.97	0.97
GrIS-16-Aero-16	02-08-2016 – 03-08-2016	Active sampling onto PC filter	19:00	8008	216	1.28	1.45
GrIS-16-Aero-21	04-08-2016 – 05-08-2016	Active sampling onto PC filter	13:20	7735	1445	0.57	0.60
GrIS-16-Aero-24	06-08-2016 – 08-08-2016	Active sampling onto PC filter	48:00	20181	962	1.00	1.12
GrIS-16-Aero-28	10-08-2016 – 11-08-2016	Active sampling onto PC filter	26:30	11165	506	0.58	0.82
GrIS-16-Aero-36	19-08-2016 – 21-08-2016	Active sampling onto PC filter	29:35	16240	303	1.16	0.68
GrIS-16-CR-8	29-07-2016	Coriolis sampling into UHQ water, filtered onto DNA filter	02:00	30000	NA	NA	NA
GrIS-16-CR-9	30-07-2016	Coriolis sampling into UHQ water, filtered onto DNA filter	03:30	63000	NA	NA	NA
GrIS-16-CR-10	31-07-2016	Coriolis sampling into UHQ water, filtered onto DNA filter	08:00	138000	NA	NA	NA
GrIS-17-Aero-1	10-06-2017 – 13-06-2017	Active sampling onto PC filter	13:43	22349	8686	0.65	0.66
GrIS-17-Aero-2	13-06-2017 – 16-06-2017	Active sampling onto PC filter	11:35	18266	3209	0.73	0.68
GrIS-17-Aero-3	16-06-2017 – 19-06-2017	Active sampling onto PC filter	15:07	9146.6	883	0.95	0.92
GrIS-17-Aero-4	22-06-2017 – 28-06-2017	Active sampling onto PC filter	17:08	14850.4	1292	1.46	1.74
GrIS-17-CR-1	06-06-2017	Coriolis sampling into UHQ water, filtered onto DNA filter	00:20	6000	NA	NA	NA

GrIS-17-CR-2	06-06-2017	Coriolis sampling into UHQ water, filtered onto PC filter	00:20	6000	347	1.09	0.93
GrIS-17-CR-3	10-06-2017	Coriolis sampling into UHQ water, filtered onto DNA filter	00:20	6000	NA	NA	NA
GrIS-17-CR-4	10-06-2017	Coriolis sampling into UHQ water, filtered onto PC filter	00:20	6000	960	1.07	0.75
GrIS-17-CR-5	11-06-2017	Coriolis sampling into UHQ water, filtered onto DNA filter	01:00	18000	NA	NA	NA
GrIS-17-CR-6	11-06-2017	Coriolis sampling into UHQ water, filtered onto PC filter	01:00	18000	15243	0.42	0.73
GrIS-17-CR-7	13-06-2017	Coriolis sampling into UHQ water, filtered onto DNA filter	02:00	36000	NA	NA	NA
GrIS-17-CR-8	13-06-2017	Coriolis sampling into UHQ water, filtered onto PC filter	02:00	36000	6623	1.04	1.00
GrIS-17-CR-9	14-06-2017	Coriolis sampling into UHQ water, filtered onto DNA filter	02:00	36000	NA	NA	NA
GrIS-17-CR-10	14-06-2017	Coriolis sampling into UHQ water, filtered onto PC filter	02:00	36000	4076	0.94	0.72

GrIS-17-CR-11	15-06-2017	Coriolis sampling into UHQ water, filtered onto DNA filter	03:00	54000	NA	NA	NA
GrIS-17-CR-12	15-06-2017	Coriolis sampling into UHQ water, filtered onto PC filter	03:00	54000	6940	0.73	0.85
GrIS-17-CR-13	16-06-2017	Coriolis sampling into UHQ water, filtered onto DNA filter	01:00	18000	NA	NA	NA
GrIS-17-CR-14	16-06-2017	Coriolis sampling into UHQ water, filtered onto PC filter	01:00	18000	5293	1.16	1.53
GrIS-17-CR-16	28-06-2017	Coriolis sampling into UHQ water, filtered onto PC filter	03:00	54000	12002	1.87	2.52

PC: polycarbonate
UHQ: ultra-high quality
NA: not applicable, specifically due to particle counts and sizes not being documented for the DNA filters

Table S3. Elemental composition determined using energy dispersive spectroscopy of mineral dust collected using the Coriolis and transferred onto polycarbonate filters (GrIS-17-CR-16), and collected directly onto aerosol filter (GrIS-17-Aero-1, GrIS-17-Aero-4).

**Weight %**	**Region in sample GrIS-17-CR-16 (n=7)**								
**Element**	**1**	**2**	**3**	**4**	**5**	**6**	**7**	**Mean (wt%)**	**SD (wt%)**
O	54.9	55.2	49.6	51.5	53.4	53.3	54.0	53.1	2.0
Na	3.9	2.8	6.4	5.5	5.6	7.3	7.1	5.5	1.7
Mg	3.0	4.8	4.0	3.5	4.1	4.4	4.3	4.0	0.6
Al	7.6	8.5	7.9	7.7	7.6	7.6	7.5	7.8	0.3
Si	22.2	18.7	22.2	22.8	19.2	19.2	19.9	20.6	1.7
K	2.1	2.9	2.7	1.8	2.1	2.0	1.6	2.2	0.5
Ca	3.8	3.3	3.3	3.4	3.3	3.0	2.7	3.3	0.3
Fe	2.5	3.8	4.0	3.7	4.7	3.1	2.9	3.5	0.7
	100.0	100.0	100.0	100.0	100.0	100.0	100.0	100.0	
**Atomic %**	**Region in sample GrIS-17-CR-16 (n=7)**								
**Element**	**1**	**2**	**3**	**4**	**5**	**6**	**7**	**Mean (at%)**	**SD (at%)**
O	68.8	69.2	63.8	65.8	67.7	66.9	67.3	67.1	1.8
Na	3.4	2.5	5.8	4.9	4.9	6.4	6.2	4.9	1.4
Mg	2.5	4.0	3.3	2.9	3.4	3.7	3.5	3.3	0.5
Al	5.7	6.3	6.1	5.8	5.7	5.7	5.5	5.8	0.3
Si	15.8	13.3	16.4	16.6	13.9	13.7	14.2	14.8	1.4
K	1.0	1.5	1.4	0.9	1.1	1.0	0.8	1.1	0.2
Ca	1.8	1.7	1.7	1.7	1.7	1.5	1.4	1.6	0.2
Fe	0.9	1.4	1.5	1.4	1.7	1.2	1.0	1.3	0.3
	100.0	100.0	100.0	100.0	100.0	100.0	100.0	100.0	
**Weight %**	**Region in sample GrIS-17-Aero-1 (n=5)**								
**Element**	1	2	3	4	5			**Mean (wt%)**	**SD (wt%)**
O	88.2	89.5	83.3	84.5	78.8			84.9	4.2
Na	1.0	0.7	1.1	1.5	3.0			1.4	0.9
Mg	0.5	0.7	0.5	1.5	1.0			0.8	0.4
Al	1.5	2.0	2.2	2.0	2.5			2.0	0.3
Si	7.2	4.6	7.5	7.0	12.3			7.7	2.8
Cl	0	1.3	1.1	1.0	1.0			0.9	0.5
K	1.0	0.7	1.1	1.5	1.0			1.0	0.3
Ca	0.5	0.7	0.5	0.5	0.5			0.5	0.1
Fe	0.0	0.0	2.7	0.5	0.0			0.6	1.2
**Atomic %**	**Region in sample GrIS-17-Aero-1 (n=5)**								
**Element**	1	2	3	4	5			**Mean (at%)**	**SD (at%)**
O	93.2	93.9	89.9	90.0	86.5			90.7	3.0
Na	0.7	0.9	0.7	1.3	2.0			1.1	0.6
Mg	0.7	0.9	0.7	1.3	0.7			0.9	0.3
Al	0.7	0.9	1.4	1.3	2.0			1.3	0.5
Si	4.1	2.6	5.1	4.7	7.4			4.8	1.7
Cl	0.0	0.9	0.7	0.7	0.7			0.6	0.3
K	0.7	0.0	0.7	0.7	0.7			0.5	0.3
Ca	0.0	0.0	0.0	0.0	0.0			0.0	0.0
Fe	0.0	0.0	0.7	0.0	0.0			0.1	0.3
**Weight %**	**Region in sample GrIS-17-Aero-4 (n=1)**								
**Element**	1	2						**Mean (wt%)**	
O	83.5	83.4						83.5	

Na	3.2	3.9						3.5	
Mg	2.1	2.2						2.2	
Al	2.7	2.8						2.7	
Si	3.7	2.8						3.2	
K	1.6	1.7						1.6	
Ca	1.1	1.1						1.1	
Ti	1.6	1.7						1.6	
Fe	0.5	0.6						0.5	
**Weight %**	**Region in sample GrIS-17-Aero-4 (n=2)**								
**Element**	1	2						**Mean (at%)**	
O	90.1	89.7						89.9	
Na	2.1	2.9						2.5	
Mg	1.4	1.5						1.4	
Al	2.1	2.2						2.2	
Si	2.1	1.5						1.8	
K	0.7	0.7						0.7	
Ca	0.7	0.7						0.7	
Ti	0.7	0.7						0.7	
Fe	0.0	0.0						0.0	

Table S4. Particulate concentrations in melted snow samples.

Sample number	Sample Collection Date	Sample type	Particulate Mass (mg)	Melt Volume (L)	Particulate Concentration ($mg \cdot L^{-1}$)
GrIS-17-22	18-Jun-17	fresh snow	1.700	0.310	5.48
GrIS-17-23	20-Jun-17	fresh snow	0.271	0.658	0.41
GrIS-17-18	07-Jun-17	weathered snow	7.498	0.73	10.27
GrIS-17-19	08-Jun-17	weathered snow	2.495	0.195	12.79
GrIS-17-20	10-Jun-17	weathered snow	3.671	0.175	20.98
GrIS-17-40	02-Jun-17	weathered snow	22.138	1.68	13.18
GrIS-17-41	02-Jun-17	weathered snow	12.410	1.43	8.68
GrIS-17-42	02-Jun-17	weathered snow	35.470	1.96	18.10
GrIS-17-43	02-Jun-17	weathered snow	15.740	1.74	9.05
GrIS-17-44	02-Jun-17	weathered snow	13.680	1.45	9.43

Table S5. Abundance of mineral phases identified using X-ray diffraction of the dust collected from snow samples in 2017. Phases were grouped into the listed classes as described in the text above.

**Sample ID**	**GrIS-17-20**	**GrIS-17-21**	**GrIS-17-23**
**Sample type**	**Weathered snow**	**Weathered snow**	**Fresh snow**
Quartz	23.6	21.4	28.7
Plagioclase feldspars	49.3	48.3	47.1
Potassium feldspars	7.7	8.3	8.9
Amphiboles	7.3	8.7	2.2
Pyroxenes	8.1	10.0	10.2
Phyllosilicates	3.8	2.7	2.7
Hydroxylapatite	0.2	0.7	0.3
Total (wt%)	100.0	100.0	100.0
rwp	13.4	14.7	16.3

Table S6. Rare earth Element (REE) concentrations (μg·g^{-1}) for mineral dust in melted snow samples collected in 2017.

Sample type	Sample ID	La	Ce	Pr	Nd	Sm	Eu	Gd	Tb	Dy	Ho	Er	Tm	Yb	Lu
Weathered snow	GrIS-17-20	0.628	0.624	0.638	0.638	0.656	0.890	0.653	0.539	0.557	0.528	0.526	0.605	0.576	0.544
Weathered snow	GrIS-17-21	0.673	0.668	0.690	0.686	0.690	0.934	0.705	0.587	0.596	0.573	0.573	0.656	0.620	0.611
Fresh snow	GrIS-17-23	0.417	0.406	0.403	0.401	0.394	0.671	0.417	0.338	0.341	0.320	0.323	0.373	0.344	0.349

Table S7. Dissolved element concentrations in melted snow and ice samples collected in 2017 measured using ICP-MS and reported in μg·L^{-1} or ng·L^{-1}, with corresponding level of detection (LOD) and level of quantification (LOQ) provided in the same units.

Sample type	Sample ID	Elemental concentration (μg·L^{-1})										
		Li	Na	Mg	Al	P	K	Ca	V	Cr	Mn	Fe
Weathered snow	GrIS-17-7	2.66E-02	3.80E+01	<LOQ	<LOD	3.65E+00	9.50E+00	1.54E+01	2.71E-01	<LOD	9.00E-02	6.28E-01
Weathered snow	GrIS-17-8	2.23E-02	2.67E+01	1.26E+00	<LOD	1.26E+00	5.68E+00	5.15E+00	1.09E-01	<LOD	1.91E-01	4.18E+01
Weathered snow	GrIS-17-18	<LOD	3.46E+01	<LOQ	<LOD	2.27E+00	2.87E+00	5.41E+00	2.11E-01	<LOD	<LOD	3.57E-01
Weathered snow	GrIS-17-19	<LOD	1.77E+01	<LOD	<LOD	1.72E+00	2.91E+00	4.30E+00	2.55E-01	<LOD	<LOQ	1.57E+00
Weathered snow	GrIS-17-20	<LOD	1.50E+01	<LOD	<LOD	2.56E+00	3.04E+00	4.40E+00	1.68E-01	<LOD	<LOD	6.07E-01
Fresh snow	GrIS-17-22	<LOD	7.18E+01	9.06E+00	<LOQ	1.47E+00	6.15E+00	1.08E+01	2.36E-01	<LOD	1.27E-01	3.32E+00
Fresh snow	GrIS-17-23	<LOD	1.29E+01	<LOD	<LOD	1.21E+00	2.86E+00	5.31E+00	2.10E-01	<LOD	<LOD	1.03E+00
Clean surface ice	GrIS-17-33	<LOD	4.67E+01	<LOQ	<LOD	1.07E+00	1.65E+01	1.02E+01	8.80E-02	<LOD	1.20E-01	1.90E+00
Clean core ice	GrIS-17-17	<LOD	1.24E+01	<LOD	<LOD	1.74E+00	7.21E+00	1.50E+01	3.03E-01	<LOD	<LOD	8.46E-01
LOD (μg·L^{-1})		5.73E-03	4.08E-01	3.10E-01	1.53E+00	1.70E-01	1.35E-01	7.84E-01	1.69E-02	1.83E-02	1.82E-02	6.10E-02
LOQ (μg·L^{-1})		1.91E-02	1.36E+00	1.03E+00	5.11E+00	5.70E-01	4.51E-01	2.61E+00	5.64E-02	6.09E-02	6.08E-02	2.03E-01
% uncertainty		2.10	3.13	1.24	2.66	4.81	3.46	2.55	3.39	0.88	1.06	2.19

Table S7 Continued.

Sample type	Sample ID	Elemental concentration (µg·L^{-1})										
		**Co**	**Ni**	**Cu**	**Zn**	**Ga**	**As**	**Rb**	**Sr**	**Ag**	**Cd**	**Pb**
Weathered snow	GrIS-17-7	<LOD	<LOD	<LOD	5.27E+00	<LOD	<LOD	<LOD	<LOD	<LOD	<LOD	<LOD
Weathered snow	GrIS-17-8	<LOD	8.33E+00	<LOD	3.80E+00	<LOD	<LOD	<LOD	<LOD	<LOD	<LOD	<LOD
Weathered snow	GrIS-17-18	<LOD	<LOD	<LOD	2.66E+00	<LOD	<LOD	<LOD	<LOD	<LOQ	<LOD	<LOD
Weathered snow	GrIS-17-19	<LOD	4.44E-01	<LOD	1.31E+00	<LOD	<LOD	<LOD	<LOD	<LOD	<LOD	<LOD
Weathered snow	GrIS-17-20	<LOD	<LOD	<LOD	3.25E+00	<LOD	<LOD	<LOD	<LOD	<LOD	<LOD	<LOD
Fresh snow	GrIS-17-22	<LOD	1.13E+00	<LOD	2.72E+00	<LOD	<LOD	<LOD	<LOD	<LOD	<LOD	<LOD
Fresh snow	GrIS-17-23	<LOD	<LOQ	<LOD	2.92E+00	<LOD	<LOD	<LOD	<LOD	<LOQ	<LOD	<LOD
Clean surface ice	GrIS-17-33	<LOD	<LOD	<LOD	3.92E+00	<LOD	<LOD	<LOD	<LOD	<LOD	<LOD	<LOD
Clean core ice	GrIS-17-17	<LOD	<LOQ	<LOD	2.97E+00	<LOD	<LOD	<LOD	<LOD	<LOD	<LOD	<LOD
LOD (µg·L^{-1})		1.44E-02	2.50E-02	3.11E-02	5.37E-02	1.68E-02	9.23E-03	2.62E-02	2.22E-02	7.97E-03	2.26E-03	1.56E-02
LOQ (µg·L^{-1})		4.80E-02	8.33E-02	1.04E-01	1.79E-01	5.61E-02	3.08E-02	8.73E-02	7.39E-02	2.66E-02	7.53E-03	5.20E-02
% uncertainty		1.29	1.16	0.99	0.97	0.79	3.79	3.63	1.29	0.91	0.50	1.24

Table S7 Continued.

Sample type	Sample ID	Elemental concentration (ng·L^{-1})										
		**Zr**	**Nb**	**Mo**	**Sn**	**Sb**	**Cs**	**Ba**	**La**	**Ce**	**Pr**	**Nd**
Weathered snow	GrIS-17-7	<LOQ	<LOD	1.18E+01	<LOD	3.07E+01	<LOD	1.44E+03	<LOQ	7.57E+01	<LOD	<LOD
Weathered snow	GrIS-17-8	6.31E+00	<LOD	<LOQ	<LOD	3.03E+01	<LOD	2.88E+02	<LOD	1.74E+01	<LOD	<LOD
Weathered snow	GrIS-17-18	<LOQ	<LOD	3.29E+00	<LOD	<LOD	<LOD	<LOD	<LOD	<LOQ	<LOD	<LOD
Weathered snow	GrIS-17-19	<LOD	<LOD	<LOQ	<LOD	<LOD	<LOD	6.32E+01	<LOQ	7.71E+00	<LOD	<LOD
Weathered snow	GrIS-17-20	7.15E+00	<LOD	7.63E+00	<LOD	<LOD	<LOD	6.97E+02	<LOQ	3.75E+01	<LOD	<LOD
Fresh snow	GrIS-17-22	<LOQ	<LOD	3.59E+00	1.56E+02	<LOD	<LOD	1.23E+02	1.15E+01	2.16E+01	<LOQ	3.31E+00
Fresh snow	GrIS-17-23	2.01E+01	<LOD	5.74E+00	<LOD	<LOD	<LOD	5.33E+02	<LOD	4.27E+01	<LOD	<LOD
Clean surface ice	GrIS-17-33	<LOQ	<LOD	3.20E+00	<LOD	<LOD	<LOD	1.15E+02	<LOD	<LOQ	<LOD	<LOQ
Clean core ice	GrIS-17-17	7.56E+00	<LOD	7.32E+00	<LOD	<LOD	<LOD	4.06E+02	<LOD	1.88E+01	<LOD	<LOD
LOD (ng·L^{-1})		1.02E+00	1.16E-01	6.93E-01	5.42E+00	4.58E+00	2.90E-01	9.11E+00	6.23E-01	1.06E+00	3.06E-01	8.64E-01
LOQ (ng·L^{-1})		3.40E+00	3.85E-01	2.31E+00	1.81E+01	1.53E+01	9.68E-01	3.04E+01	2.08E+00	3.52E+00	1.02E+00	2.88E+00
% uncertainty		1.95	1.97	2.19	3.83	2.91	1.47	6.04	1.34	1.89	0.86	2.34

Table S8. Comparison of dissolved phosphorus concentrations measured in melted snow and ice samples using ICP-MS and LWCC, reported in $\mu g \cdot L^{-1}$, with corresponding level of detection (LOD) and level of quantification (LOQ) provided in the same units. LOD is not available for the LWCC results.

**Sample type**	**Sample ID**	**P measured by ICP-MS ($\mu g \cdot L^{-1}$)**	**P measured by LWCC ($\mu g \cdot L^{-1}$)**
Weathered snow	GrIS-17-7	3.65	0.98
Weathered snow	GrIS-17-8	1.26	1.28
Weathered snow	GrIS-17-18	2.27	1.33
Weathered snow	GrIS-17-19	1.72	1.03
Weathered snow	GrIS-17-20	2.56	2.16
Fresh snow	GrIS-17-22	1.47	1.81
Fresh snow	GrIS-17-23	1.21	1.07
Clean surface ice	GrIS-17-33	1.07	0.76
Clean core ice	GrIS-17-17	1.74	0.27
LOD ($\mu g \cdot L^{-1}$)		0.17	n.a.
LOQ ($\mu g \cdot L^{-1}$)		0.57	0.37
% uncertainty		4.81	5.1

Table S9. Measured pH, conductivity, and total dissolved solids for melted snow and ice samples collected in 2017.

Sample type	Sample ID	pH	Conductivity ($\mu S \cdot cm^{-1}$)	Total dissolved solids (ppm)
Weathered snow	GrIS-17-7	5.56	1.77	No measurement
Weathered snow	GrIS-17-8	5.36	1.877	1.419
Weathered snow	GrIS-17-18	5.6	1.53	1.251
Weathered snow	GrIS-17-19	5.54	1.344	1.183
Weathered snow	GrIS-17-20	5.56	1.329	1.168
Fresh snow	GrIS-17-22	4.55	6.851	3.84
Fresh snow	GrIS-17-23	4.45	2.745	1.88
Clean ice	GrIS-17-33	5.26	3.457	2.189

Table S10. Monthly snowfall in mmWE for the SW Greenland drainage basin from MAR v3.11.2. Estimated snowfall and the measured particle mass loading in fresh snow (2.9 $mg \cdot kg^{-1}$, n=2) were used to calculate monthly and annual (see main text) particle flux values for particle deposition via snowfall.

Month	Estimated monthly snowfall (mmWE)	Calculated monthly particle flux ($mg \cdot m^{-2}$)
January	13.6	40.0
February	11.8	34.7
March	16.9	49.7
April	25.0	73.6
May	28.7	84.7
June	18.0	53.1
July	25.0	73.6
August	26.8	79.0
September	48.0	141.6
October	41.3	121.9
November	33.8	99.6
December	21.2	62.4

Captions for supplemental movies (S1 – S14) and datasets (S1 – S5) deposited to Zenodo (citations listed below), and the microbial community datasets (S6 and S7) deposited to NCBI under BioProject PRJNA1287549:

McQuaid, J., & McCutcheon, J. (2025, December 19). Black and Bloom Work Package 2 - Particulates - FLEXPART Movies. Zenodo. https://doi.org/10.5281/zenodo.17990403

McQuaid, J., & McCutcheon, J. (2025). Black and Bloom Work Package 2 - Particulates - Aerosol and Meteorological data (Version 1) [Data set]. Zenodo. https://doi.org/10.5281/zenodo.17985533

Movie S1. Animated FLEXPART potential emission sensitivity (PES) outputs for 0 – 100 m above ground level (AGL) launched from the campaign site between 10/07/2016 – 22/08/2016.

Movie S2. Animated FLEXPART PES outputs for 0 – 500 m AGL launched from the campaign site between 10/07/2016 – 22/08/2016.

Movie S3. Animated FLEXPART PES outputs for 0 – 1000 m AGL launched from the campaign site between 10/07/2016 – 22/08/2016.

Movie S4. Animated FLEXPART PES outputs for 0 – 2500 m AGL launched from the campaign site between 10/07/20216 – 22/08/2016.

Movie S5. Animated FLEXPART PES outputs for 0 – 5000 m AGL launched from the campaign site between 10/07/20216 – 22/08/2016.

Movie S6. Animated FLEXPART PES outputs for 0 – 10000 m AGL launched from the campaign site between 10/07/2016 – 22/08/2016.

Movie S7. Animated FLEXPART PES outputs for 500 – 10000 m AGL launched from the campaign site between 10/07/2016 – 22/08/2016.

Movie S8. Animated FLEXPART PES outputs for 0 – 100 m AGL launched from the campaign site between 30/05/20217 – 01/07/2017.

Movie S9. Animated FLEXPART PES outputs for 0 – 500 m AGL launched from the campaign site between 30/05/20217 – 01/07/2017.

Movie S10. Animated FLEXPART PES outputs for 0 – 1000 m AGL launched from the campaign site between 30/05/20217 – 01/07/2017.

Movie S11. Animated FLEXPART PES outputs for 0 – 2500 m AGL launched from the campaign site between 30/05/20217 – 01/07/2017.

Movie S12. Animated FLEXPART PES outputs for 0 – 5000 m AGL launched from the campaign site between 30/05/20217 – 01/07/2017.

Movie S13. Animated FLEXPART PES outputs for 0 – 10000 m AGL launched from the campaign site between 30/05/20217 – 01/07/2017.

Movie S14. Animated FLEXPART PES outputs for 500 – 10000 m AGL launched from the campaign site between 30/05/20217 – 01/07/2017.

Dataset S1. Meteorology data collected using a WS-GP1 weather station (Delta T Devices Ltd) during the 2017 field campaign.

Dataset S2. Particle count data collected using an Alphasense-N2 optical particle counter (OPC) during the 2016 field campaign.

Dataset S3. Particle count data collected using an Alphasense-N2 optical particle counter (OPC) during the 2017 field campaign.

Dataset S4. Particle flux calculation summary for the 2016 and 2017 field campaigns.

Calculations completed as described in the methods using the meteorology and optical particle counter data.

Dataset S5. Aerosol particle size measurement summary for aerosols collected on filters during the 2016 and 2017 field campaigns. Measurements made using scanning electron microcopy and ImageJ software. Sample IDs correspond to those listed in Table S2, and those including 'CR' denote samples collected using the Coriolis® μ air sampler.

Dataset S6. Rarefied operational taxonomic unit (OTU) data from the 16S amplicon sequencing results for the air and snow samples.

Dataset S7. Rarefied operational taxonomic unit (OTU) data from the 18S amplicon sequencing results for the air and snow samples.

SI References

1. Alphasense Ltd. (2015) Alphasense Unser Manual OPC-NS Optical Particle Counter. p 15.
2. S. Sousan, K. Koehler, L. Hallett, T. M. Peters, Evaluation of the Alphasense optical particle counter (OPC-N2) and the Grimm portable aerosol spectrometer (PAS-1.108). *Aerosol Science and Technology* **50**, 1352-1365 (2016).
3. L. R. Crilley *et al.*, Evaluation of a low-cost optical particle counter (Alphasense OPC-N2) for ambient air monitoring. *Atmos. Meas. Tech.* **11**, 709-720 (2018).
4. Bruker AXS (2004) Topas V. 3.0: General Profile and Structure Analysis Software for Powder Diffraction Data. (Bruker AXS, Germany).
5. R. W. Cheary, A. Coelho, A fundamental parameters approach to X-ray line-profile fitting. *Journal of Applied Crystallography* **25**, 109-121 (1992).
6. H. M. Rietveld, A profile refinement method for nuclear and magnetic structures. *Journal of Applied Crystallography* **2**, 65-71 (1969).
7. D. L. Bish, S. A. Howard, Quantitative phase analysis using the Rietveld method. *Journal of Applied Crystallography* **21**, 86-91 (1988).
8. R. Hill, C. Howard, Quantitative phase analysis from neutron powder diffraction data using the Rietveld method. *J. Appl. Crystallogr.* **20**, 467-474 (1987).
9. J. McCutcheon *et al.*, Mineral phosphorus drives glacier algal blooms on the Greenland Ice Sheet. *Nature Communications* **12**, 570 (2021).
10. E. Bolyen *et al.*, Reproducible, interactive, scalable and extensible microbiome data science using QIIME 2. *Nature Biotechnology* **37**, 852-857 (2019).
11. B. J. Callahan *et al.*, DADA2: High-resolution sample inference from Illumina amplicon data. *Nature Methods* **13**, 581-583 (2016).
12. C. Quast *et al.*, The SILVA ribosomal RNA gene database project: improved data processing and web-based tools. *Nucleic acids research* **41**, D590-D596 (2013).
13. M. S. Robeson *et al.*, RESCRIPt: Reproducible sequence taxonomy reference database management. *PLoS computational biology* **17**, e1009581 (2021).
14. M. R. McLaren, Silva SSU taxonomic training data formatted for DADA2 (Silva version 138). *(No Title)* (2020).
15. R Core Team (2021) R: A language and environment for statistical computing. R Foundation for Statistical Computing. (Vienna, Austria).

16. P. J. McMurdie, S. Holmes, phyloseq: an R package for reproducible interactive analysis and graphics of microbiome census data. *PloS one* **8**, e61217 (2013).
17. A. Kassambara, ggpubr:'ggplot2'based publication ready plots. *R package version 3.3.5* (2020).
18. K. D. Froyd *et al.*, A new method to quantify mineral dust and other aerosol species from aircraft platforms using single-particle mass spectrometry. *Atmos. Meas. Tech.* **12**, 6209-6239 (2019).
19. E. Andreas (1989) Thermal and Size Evolution of Sea Spray Droplets. (Defense Technical Information Center), pp 1-38.
20. United States Environmental Protection Agency (2000) Control of Particulate Matter Emissions - Student Manual, 3rd ed. (ICES Ltd. The Multimedia Group), p 358.
21. W. A. Hoppel, G. M. Frick, J. W. Fitzgerald, Surface source function for sea-salt aerosol and aerosol dry deposition to the ocean surface. *Journal of Geophysical Research: Atmospheres* **107**, AAC 7-1-AAC 7-17 (2002).
22. W. A. Hoppel, P. F. Caffrey, G. M. Frick, Particle deposition on water: Surface source versus upwind source. *Journal of Geophysical Research* **110**, D10206 (2005).
23. Z. Zou, D. Zhao, B. Liu, J. A. Zhang, J. Huang, Observation-based parameterization of air-sea fluxes in terms of wind speed and atmospheric stability under low-to-moderate wind conditions. *J Geophys Res Oceans* **122**, 4123-4142 (2017).
24. D. H. Hagan, J. H. Kroll, Assessing the accuracy of low-cost optical particle sensors using a physics-based approach. *Atmos. Meas. Tech.* **13**, 6343-6355 (2020).
25. J. J. Lin, K. E. Noll, T. M. Holsen, Dry Deposition Velocities as a Function of Particle Size in the Ambient Atmosphere. *Aerosol Science and Technology* **20**, 239-252 (1994).
26. S. Mariraj Mohan, An overview of particulate dry deposition: measuring methods, deposition velocity and controlling factors. *International Journal of Environmental Science and Technology* **13**, 387-402 (2016).
27. A. J.-M. Bory, P. E. Biscaye, A. M. Piotrowski, J. P. Steffensen, Regional variability of ice core dust composition and provenance in Greenland. *Geochemistry Geophysics Geosystems* **4** (2003).
28. I. G. M. Wientjes, R. S. W. Van de Wal, G. J. Reichart, A. Sluijs, J. Oerlemans, Dust from the dark region in the western ablation zone of the Greenland ice sheet. *The Cryosphere* **5**, 589-601 (2011).
29. N. Tepe, M. Bau, Distribution of rare earth elements and other high field strength elements in glacial meltwaters and sediments from the western Greenland Ice Sheet: Evidence for different sources of particles and nanoparticles. *Chemical Geology* **412**, 59-68 (2015).
30. T. F. D. Nielsen, S. M. Jensen, K. Secher, K. K. Sand, Distribution of kimberlite and aillikite in the Diamond Province of southern West Greenland: A regional perspective based on groundmass mineral chemistry and bulk compositions. *Lithos* **112**, 358-371 (2009).

31. M. Ferrat *et al.*, Improved provenance tracing of Asian dust sources using rare earth elements and selected trace elements for palaeomonsoon studies on the eastern Tibetan Plateau. *Geochimica et Cosmochimica Acta* **75**, 6374-6399 (2011).
32. M. van der Does, A. Pourmand, A. Sharifi, J.-B. W. Stuut, North African mineral dust across the tropical Atlantic Ocean: Insights from dust particle size, radiogenic Sr-Nd-Hf isotopes and rare earth elements (REE). *Aeolian Research* **33**, 106-116 (2018).
33. B. Vandecrux *et al.*, The historical Greenland Climate Network (GC-Net) curated and augmented level-1 dataset. *Earth Syst. Sci. Data* **15**, 5467-5489 (2023).
